# Climate and Land‐Use Changes Predicted to Jointly Drive Soil Fungal Diversity Losses in One‐Third of North American Coniferous Forests

**DOI:** 10.1111/gcb.70598

**Published:** 2025-11-08

**Authors:** Wenqi Luo, Kabir G. Peay, Thiago Gonçalves‐Souza, Peter B. Reich, Donald R. Zak, Kai Zhu

**Affiliations:** ^1^ Institute for Global Change Biology and School for Environment and Sustainability University of Michigan Ann Arbor Michigan USA; ^2^ Department of Biology Stanford University Stanford California USA; ^3^ Department of Earth System Science Stanford University Stanford California USA; ^4^ Department of Ecology and Evolutionary Biology University of Michigan Ann Arbor Michigan USA; ^5^ Department of Forest Resources University of Minnesota St. Paul Minnesota USA

**Keywords:** climate change, habitat loss, land‐use change, soil fungal diversity, species–area relationship

## Abstract

Soil fungi underpin key ecosystem functions but face increasing threats from climate and land‐use changes, with their future impacts remaining unclear. This uncertainty is exacerbated by limited large‐scale data and the challenge of quantifying and comparing both factors at comparable spatial scales. By leveraging two continental‐scale sampling networks in North America and applying stacked species distribution models combined with countryside species–area relationship frameworks, we assessed the impacts of climate and land‐use change on soil fungal diversity and identified regions affected by both factors across four biomes. We projected climate and land‐use change by incorporating shared socioeconomic pathways (SSPs) and associated greenhouse gas–induced radiative forcing, focusing on moderate‐ (SSP2–4.5) and high‐emission (SSP5–8.5) scenarios. Climate change typically led to both diversity losses and gains, particularly in coniferous forests and among arbuscular mycorrhizal (AM) fungi. Land‐use change predominantly caused diversity losses under SSP2–4.5, especially in broadleaf‐mixed forests and for ectomycorrhizal (EM) fungi, with these effects diminished under SSP5–8.5 due to minimal land‐use changes. Across emission scenarios, both factors were predicted to cause widespread diversity losses in coniferous forests (whole‐community, EM fungi, and soil saprotrophs) and grasslands (AM fungi and plant pathogens) while promoting gains in broadleaf‐mixed forests (whole‐community, EM fungi, and saprotrophs) and coniferous forests (AM fungi and pathogens). These results support the need for biome‐ and guild‐specific fungal conservation planning under global change.

## Graphical abstract

This study assessed the potential impacts of climate and land‐use change on soil fungal diversity across four North American biomes. We found that climate change typically caused both diversity losses and gains, particularly in coniferous forests and among arbuscular mycorrhizal fungi, whereas land‐use change mainly reduced diversity, especially in broadleaf‐mixed forests and for ectomycorrhizal fungi. Despite guild‐specific responses, both drivers were projected to cause widespread whole‐community fungal losses in coniferous forests and grasslands, while promoting gains in broadleaf‐mixed and coniferous forests. These results emphasize the need for biome‐ and guild‐specific strategies to conserve fungal diversity under global change.

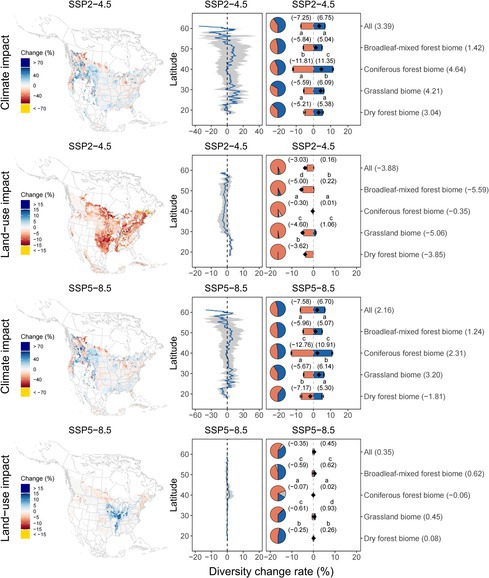

## 抽象的

土壤真菌在维持生态系统功能中发挥着重要作用，但正面临气候变化和土地利用变化的双重威胁，其未来影响仍不清楚。这种不确定性主要源于缺乏大尺度观测数据，以及难以在可比空间尺度上同时量化和比较这两类因子的效应。基于北美大陆尺度的两个采样网络，结合物种分布模型与“乡村物种–面积关系”框架，本研究系统评估了气候变化和土地利用变化对北美四大生物群系中土壤真菌多样性的影响，并识别了可能同时受这两类因子作用的区域。通过引入共享社会经济路径(SSPs)及其对应的温室气体辐射强度，研究重点模拟了中等(SSP2–4.5)和高排放(SSP5–8.5)两种情景下的气候与土地利用变化。结果表明，气候变化通常同时导致真菌多样性的增加和减少，尤其显著影响针叶林区的丛枝菌根(AM)真菌；而土地利用变化在SSP2–4.5情景下主要导致真菌多样性的减少，尤其显著影响阔叶混交林区的外生菌根(EM)真菌，但在SSP5–8.5情景下，由于土地利用变化幅度有限，该效应微弱。在两种排放情景下，气候变化和土地利用变化均被预测将在针叶林(整体群落、EM真菌和土壤腐生真菌)和草地(AM真菌和植物病原真菌)生物群系中导致广泛的多样性丧失，而在阔叶混交林(整体群落、EM真菌和腐生真菌)以及针叶林(AM真菌和病原真菌)生物群系中则可能促进多样性。在全球变化背景下，本研究强调了制定兼顾生物群系类型和真菌功能群特征的真菌保护与管理策略的必要性。

## 1 Introduction

Climate and land‐use changes are reshaping biodiversity worldwide with profound implications for ecosystem functions. While recent efforts have examined how these changes affect regional or global macroorganism diversity (Marques et al. 2019; Pereira et al. 2024), few studies have focused on microorganisms like soil fungi, largely due to limited large‐scale data and the challenges of quantifying climate and land‐use effects at comparable spatial scales.

Soil fungi are essential ecosystem components, serving as decomposers, mutualists, and pathogens that regulate nutrient cycling and plant dynamics (Case et al. 2025). Many studies have identified key climatic factors, such as temperature and precipitation, as critical drivers of soil fungal distributions (Větrovský et al. 2019; Qin et al. 2023). These findings provide a foundation for projecting fungal community shifts under climate change scenarios (Qin et al. 2023). Similarly, land‐use change from natural (e.g., forests) to human‐dominated land systems (e.g., croplands) alters fungal diversity and composition (Labouyrie et al. 2023; Qu et al. 2024). Yet, few studies have examined and compared the potentially distinct effects of climate and land‐use changes on soil fungal diversity, primarily due to the challenges of quantifying both effects at comparable spatial scales (Newbold 2018). Climate impacts are often evaluated by modeling changes in climate suitability over time across broad landscapes (Steidinger et al. 2020; Van Nuland et al. 2024) (e.g., 10 × 10 km grid cells), whereas land‐use impacts are frequently evaluated by comparing fungal community diversity and composition between paired impacted and undisturbed control sites at fine, soil core‐level scales (Labouyrie et al. 2023, Qu et al. 2024). This spatial mismatch limits our understanding of how landscape‐scale land‐use change affects fungal diversity and hinders assessments of spatial overlap between climate and land‐use effects at broader scales.

One potential way to quantify landscape‐scale land‐use change effects on soil fungal diversity is to scale diversity from small sampling units to landscape scales using the species–area relationship (SAR), which describes how species number increases with sampling area (i.e., diversity scaling; García Martín and Goldenfeld 2006). The SAR is often modeled with a power‐law function: *S* = *cA* ^*z*^, where *S* is the species number, *A* is the area, *c* is a parameter that indicates baseline fungal diversity (i.e., expected species number within unit area), and *z* is a parameter that denotes the rate at which species increase with sampling area (i.e., scaling slope). Since SAR links diversity and area, landscape‐scale diversity losses and gains (i.e., diversity change rate) can be estimated by examining changes in the effective habitat area under climate and land‐use changes (Thomas et al. 2004). For example, extending the classic SAR, the countryside SAR models how compositional changes of land systems within a landscape affect biodiversity by accounting for species' relative affinity for different land systems (Pereira et al. 2024). Relative affinity reflects species preference for a modified system compared to a natural system and can be inferred from the ratio of diversity measured within each system at local scales (Gerstner et al. 2017). Thus, the expansion of human‐dominated land systems, for example, may lead to landscape‐scale diversity losses due to reduced effective habitat area if species show a lower relative affinity for these systems (Pereira et al. 2014). Despite its growing popularity in macroecology (Pereira et al. 2014; Gerstner et al. 2017; Marques et al. 2019), few studies have applied the countryside SAR framework to model land‐use‐driven fungal diversity change. This is largely due to the challenges of quantifying how fungal diversity scales with habitat area and how such a scaling relationship varies with fungal guilds and regional environmental gradients (Green and Bohannan 2006; Peay et al. 2007; Zhou et al. 2008). Addressing these gaps requires large‐scale and standardized sampling campaigns to construct soil fungal SARs and model their potential spatial variabilities (Tu et al. 2016).

Examining and comparing the impacts of climate and land‐use changes on fungal diversity is complicated by their interplay with ecoregions. Boreal and temperate broadleaf‐mixed forest biomes are experiencing comparatively faster warming than the global average (Meehl et al. 2015), with many fungal species in these biomes predicted to occupy a narrow climate niche (Qin et al. 2023). These dynamics suggest potentially greater vulnerability to climate‐driven diversity losses in these regions. Similarly, temperate grassland and broadleaf‐mixed forest biomes are projected to face more extensive cropland expansion, driven by their higher agricultural efficiency and human habitability (Chen et al. 2022), suggesting potentially greater land‐use‐driven diversity change in these regions. Assessing climate and land‐use changes among biomes is therefore essential for a more holistic understanding of their effects on fungal diversity.

Finally, fungal diversity responses to climate and land‐use changes may vary across fungal guilds. Globally, common mycorrhizal fungi generally occupy narrower climate niches than plant pathogens and soil saprotrophs (Tedersoo et al. 2022; Baldrian et al. 2022). In contrast, plant pathogen abundances have been shown to increase under warming (Delgado‐Baquerizo et al. 2020). Fungal guild type may affect diversity responses to land‐use change through two possible mechanisms. First, the higher dependency of mycorrhizal fungi on living plants may result in more pronounced changes in diversity per unit of land‐use change (Van Nuland et al. 2024). Second, the lower dispersal capacity of mycorrhizal fungi compared to plant pathogens and free‐living saprotrophs may increase spatial heterogeneity among fungal communities (Brown and Hovmøller 2002). Consequently, land‐use change may have a stronger negative effect on mycorrhizal fungi due to both higher per‐unit diversity losses and steeper diversity scaling slopes. In contrast, plant pathogens may be less affected, due to their stress‐induced dormancy and the potentially increased transmission among cropland monocultures (Makiola et al. 2019). Together, these findings suggest that mycorrhizal fungi may be more sensitive to both climate and land‐use changes than plant pathogens, but this hypothesis remains untested.

**FIGURE 1:**
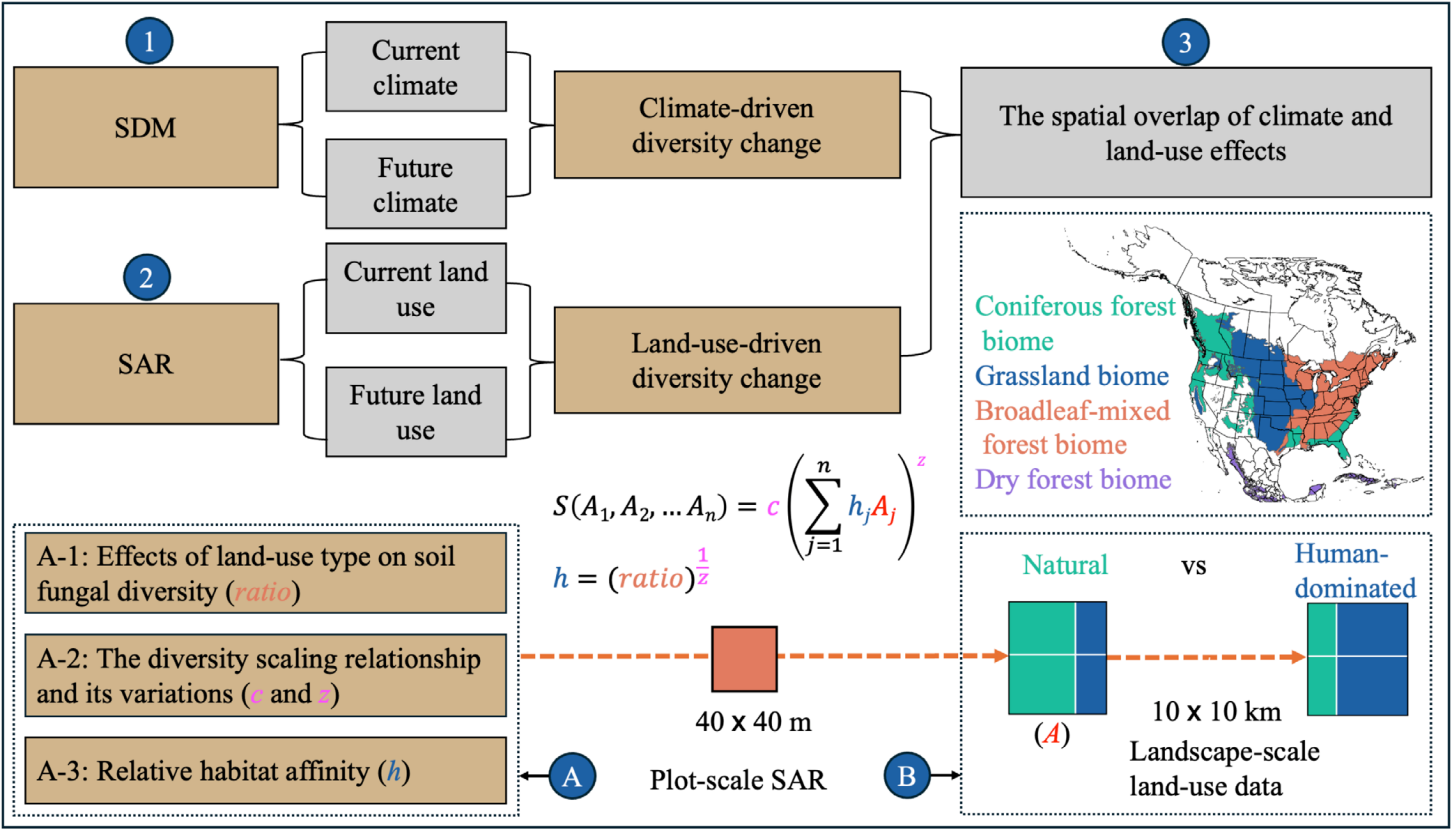
Methodological overview of the analyses, which includes the assessment of the potentially distinct effect of climate and land‐use changes on soil fungal diversity (1–2) and their spatial overlap across four biomes in North America as shown on the map (3). The individual effects of climate and land‐use change were assessed by examining how each factor drove fungal diversity losses and gains, using estimates from current and future climate or land‐use scenarios through species distribution modeling and the countryside SAR framework, respectively. The countryside SAR framework, as indicated by the equations, was parameterized with four steps, as outlined in boxes A and B. First, we determined the gamma diversity ratio (i.e., total taxonomic diversity for a given sampling effort) between human‐dominated plots and their paired natural plots for each biome (A‐1). Next, we constructed the SAR at the 40 × 40 m plot scale to estimate baseline fungal diversity (i.e., *c* parameter) and scaling slopes, and examined the spatial variability of the scaling slopes (A‐2). Based on the diversity ratio and scaling slope from steps (A‐1) and (A‐2), we then determined the species' relative affinity for the human‐dominated versus natural land systems (A‐3). Finally, we parameterized the countryside SAR framework with biome‐specific species relative affinities, baseline fungal diversity, scaling slopes, and landscape‐scale land‐use data that includes information on human‐dominated and natural land areas within each 10 × 10 km grid cell (B). The key parameters determined at each step are shown in parentheses and are color‐coded to match those in the equations.

Here, using two continental‐scale sampling networks comprising over 6300 soil samples from 128 sites encompassing over 500 plots, we examined and compared the potentially distinct effects of climate and land‐use changes on landscape‐scale soil fungal diversity across four North American biomes with three key analyses (Figure 1; Figure S1). First, building on our recent work identifying key climate variables driving current fungal distributions (Steidinger et al. 2020; Qin et al. 2023; Van Nuland et al. 2024), we investigated how plot‐scale land‐use change from natural to human‐dominated systems affected soil fungal taxonomic diversity in each biome (Table S1). The fungal diversity ratio between human‐dominated and natural systems helps infer species' relative affinity for each system. Second, to scale the effects of land‐use change from plot to landscape scales, we constructed 469 plot‐scale fungal SARs and examined their variations across fungal guilds and environmental gradients. This approach establishes a framework for estimating guild‐ and biome‐specific diversity scaling slopes by accounting for their potential variations along broad environmental gradients. Finally, with current climate and land‐use data quantified at the 10 × 10 km grid cell and their projections under two global change scenarios, we applied species distribution modeling (SDM) and the countryside SAR framework to quantify fungal diversity change driven by each factor, and identified the spatial overlap of both effects. Our projections integrated both the Shared Socioeconomic Pathways (SSPs) framework and their associated greenhouse gas–induced radiative forcing. These include a moderate greenhouse gas emission scenario (SSP2–4.5), which assumes expanded biofuel croplands to partially mitigate climate change, and a high greenhouse gas emission scenario (SSP5–8.5), characterized by intensive fossil fuel use that drives global economic growth but exacerbates climate change (Chen et al. 2022; Pang et al. 2024). Encompassing both scenarios allows for a more comprehensive exploration of potential fungal diversity changes.

Generally, we hypothesized that both climate and land‐use changes would primarily drive whole‐community fungal diversity losses by disrupting co‐evolved species–environment interactions, with these effects depending on emission scenarios. Climate‐driven diversity change would be more pronounced under SSP5–8.5 due to increased warming from higher carbon emissions. In contrast, land‐use‐driven diversity change would be more pronounced under SSP2–4.5 due to continued agricultural expansion, but less so under SSP5–8.5, where increased educational investments and social capital promote sustainable land use (Pang et al. 2024). For individual guilds, we hypothesized that both climate and land‐use changes would exert a stronger negative effect on mycorrhizal fungi than on plant pathogens, whose diversity may even be enhanced by both factors. Concerning spatial patterns, we hypothesized that climate‐driven diversity change would prevail in northern coniferous and broadleaf‐mixed forest biomes, whereas land‐use‐driven diversity change would prevail in grassland and broadleaf‐mixed forest biomes. Consequently, the spatial overlap of both factors driving diversity change would mainly occur in broadleaf‐mixed forest biomes.

## 2 Materials and Methods

### 2.1 Species and Fungal Guild Data

Soil samples were derived from two ecological networks: the Dimensions of Biodiversity of Ectomycorrhizal Fungi (DoB‐Fun) network, which includes 68 sites distributed across North American forests dominated by Pinaceae, and the National Ecological Observatory Network (NEON), which includes 47 terrestrial sites distributed across all major eco‐climatic zones in the United States (Qin et al. 2023) (Figure S1). These two networks are broadly similar in their sampling designs and species identification methods: soil samples were collected from 40 × 40 m plots and separated into organic and mineral layers. Soil sampling for NEON sites occurs annually at a minimum of one site per domain and once (sites with short growing seasons) to three times per year (sites with longer growing seasons). Soil samples were collected based on predetermined and randomly assigned locations using a 5.1 ± 1.3 cm inner diameter coring device to a maximum depth of 30 cm. We compiled data from all NEON samples collected from 2016 through 2018 (National Ecological Observatory Network 2021). Soil sampling for DoB‐Fun sites occurred from 2011 through 2013. Soil samples were collected at 13 points per plot using a coring device 7.6 cm in diameter to a depth of 14 cm.

Details about sampling and DNA extraction were described in previous studies (Talbot et al. 2014). Briefly, DNA was extracted from all soil samples, and the ITS1 region of the fungal internal transcribed spacer was targeted for amplification (Schoch et al. 2012) using the ITS1F–ITS2 primer set (Smith and Peay 2014). Sequencing was primarily performed on the Illumina MiSeq platform for all NEON samples and most DoB‐Fun samples, with the remaining DoB‐Fun samples sequenced using 454 pyrosequencing. Illumina reads were processed with the DADA2 workflow (Callahan et al. 2016) applying quality control thresholds of maxN = 0, maxEE = 8, truncQ = 2, and minLen = 50. Because DADA2 is less suited for 454 data, those reads were handled separately with QIIME and USEARCH (Edgar 2010), applying sequence length limits of 350–1200 bp, a maximum homopolymer length of 10 bp, and a maximum barcode error rate of 1.5. Due to computational constraints, denoised sequences from different sequencing runs could not be combined directly. To facilitate cross‐sequencing platform comparisons, all denoised reads were clustered into species‐level OTUs at 97% sequence identity using VSEARCH (Rognes et al. 2016), and only OTUs represented by more than 25 reads were retained for downstream analyses. Combining data sets generated from these two sampling networks yielded 7749 soil samples with 67,769 taxa from 113 sites. Fungal taxa were assigned to different trophic guilds with the FungalTraits database (Põlme et al. 2020), including the four major guilds considered in subsequent analyses: ectomycorrhizal (EM) fungi, arbuscular mycorrhizal (AM) fungi, soil saprotrophs, and plant pathogens.

### 2.2 Climate and Soil Data and Climate Projections

Current climate and soil variables were quantified at both the 40 × 40 m plot scale and the 10‐min degree pixel scale (10 × 10 km) to model the spatial variation of diversity scaling slopes and species distributions, respectively. Climate data was extracted from 1970 through 2000 with WorldClim v2 (Fick and Hijmans 2017) while soil data was extracted from the World Soil Information Service, updated in 2023 (Grunwald et al. 2011). At the plot scale, 12 climate and soil variables with a correlation coefficient below 0.75 were included: mean annual temperature (MAT), precipitation of the warmest quarter (PWQ), precipitation seasonality (Pre.seas.), temperature seasonality (Temp.seas.), mean annual precipitation (MAP), mean temperature of the wettest quarter (MTWQ), mean diurnal range (MDR: mean of monthly (max temperature‐min temperature)), soil sand content (Sand), cation exchange capacity (CEC), soil total carbon (SoilC), soil moisture, and soil pH.

At the pixel scale, seven climate and soil variables identified to be important in predicting fungal diversity were included as rasters: MAT, the quadratic term of MAT (MAT^2^), MAP, the quadratic term of MAP (MAP^2^), Temp.seas., pH, and SoilC (Qin et al. 2023). Soil samples were then aggregated into the nearest climate raster cells, resulting in 128 “operational” sites. Following the Shared Socioeconomic Pathways (SSPs) framework and their associated greenhouse gas–induced radiative forcing, two climate projections for 2100 were generated, representing moderate (SSP2–4.5) and high greenhouse gas emission scenarios (SSP5–8.5), respectively (Melillo et al. 2014). Briefly, SSP2–4.5 represents a “middle of the road” scenario that follows historical patterns of socioeconomic development and is characterized by extensive agricultural expansion, particularly biofuel‐related crops as a fossil fuel alternative to alleviate carbon emissions. SSP5–8.5 represents a “fossil fueled development” scenario that asserts transnational investments in education and social capital through heavy exploitation of fossil fuels, facilitating sustainable land‐use practices but exacerbating carbon emissions (Chen et al. 2022). Since future soil projections were unavailable, soil variables were assumed to be unchanged in the modeling projections.

### 2.3 Land‐Use Type, Classifications, and Projections

Land‐use type was quantified or classified both at the 40 × 40 m plot and 10 × 10 km pixel scales. The plot‐scale land‐use data were derived from the National Land Cover Database (NLCD) and included ten explicit classifications, which were broadly reclassified as natural (e.g., grassland and forest) and human‐dominated land systems (e.g., cultivated crops and pasture). This resulted in 45 human‐dominated plots included in subsequent analyses, spanning nine NEON sites across four biomes: temperate broadleaf and mixed forests (hereafter referred to as broadleaf‐mixed forest biome), temperate coniferous forests (coniferous forest biome), temperate grasslands (grassland biome), savannas and shrublands, and tropical and subtropical dry broadleaf forests (Olson et al. 2001) (dry forest biome; Table S1; Figure S1). To compare fungal diversity between natural and human‐dominated land systems, we paired each human‐dominated plot with a number of adjacent natural plots, ensuring that the paired plots had the same baseline historical land‐use type, which was determined with a recently published dataset (Li et al. 2023) (Note S1; Table S1). The pairing resulted in two soil sample collections that were used for the comparison of fungal diversity: one from a single human‐dominated plot and the other from multiple paired natural plots.

The pixel‐scale land‐use types were determined using a globally projected land‐use dataset at 0.05° resolution, spanning 2015–2100. This dataset was produced using the Global Change Analysis Model (GCAM) and a land‐use spatial downscaling model, under five SSPs and four Representative Concentration Pathway scenarios (Chen et al. 2022). Each pixel included 32 plant‐functional‐based land‐use types, which were reclassified into two broad categories of natural (e.g., forest and grassland) and human‐dominated land systems (e.g., bioenergy croplands). For this study, two land‐use projections for 2100 were selected, representing a moderate‐emission (SSP2–4.5) and a high‐emission scenario (SSP5–8.5), for which corresponding future climate data were derived.

### 2.4 Parameterizing the Countryside SAR Framework With Species Relative Habitat Affinity and Species–Area Relationship

The countryside SAR framework models how diversity scales with land area in a landscape with different habitats (e.g., land‐use types), where species may show varying affinities. Total species taxonomic diversity in a landscape was estimated with the formula: $S\left(A_{1},A_{2},\ldots A_{n}\right)=c{\left(\sum_{j=1}^{n}h_{j}A_{j}\right)}^{z}$, where *n* is the number of land‐use types, *h*_*j*_ indicates species relative affinity for land‐use type *j*, and *A*_*j*_ indicates area of land‐use type *j*. Theoretical studies have shown that species relative affinity for the modified habitat type *j* compared to their native habitats can be estimated with the formula: $h_{j}={\left(\frac{S_{j}}{S_{0}}\right)}^{\frac{1}{z}}$, where *S*_*j*_ and *S*_0_ represent local‐scale diversity observations (e.g., at the plot scale) in modified and native habitats, respectively, and *z* represents the diversity scaling slope (Gerstner et al. 2017) (Note S2).

To estimate species relative affinity for human‐dominated land systems, we first determined the gamma diversity for each human‐modified plot and its paired natural plots using two sample collections, deriving a diversity ratio. As described in section (2.3), since there were typically more soil samples collected in the natural plots, gamma diversity was estimated with rarefaction based on the sample size of the human‐dominated plots. Species relative affinity for human‐dominated land systems was then quantified by raising the diversity ratio to the power of the scaling slope, while species relative affinity for natural land systems was set to one.

To estimate the *c* parameters (i.e., expected fungal richness per unit area) and scaling slopes, we construct a SAR for each 40 × 40 m plot. Briefly, we estimated fungal gamma diversity at four sampling scales of 100, 400, 900, and 1600 m^2^ by randomly sampling one, four, nine, and 16 soil samples at each scale, respectively, and fit a linearized SAR with the formula: log(*S*) = log(*c*) + *z*log(*A*) for 469 plots with adequate soil samples (Figure S11). Like the power‐law model, the slope (*z*) in the linearized SAR model represents how rapidly fungal richness increases with area. The intercept corresponds to the log‐transformed *c* parameter, which can be interpreted as the expected fungal richness per unit area. Because *c* is context dependent, it should be interpreted only within the ecological, taxonomic, and methodological framework in which it was estimated. In this study, where fungal diversity was assessed per square meter, *c* can be interpreted as the extrapolated fungal richness in 1 m^2^.

The countryside SAR framework was parameterized with species relative affinity, *c* parameters, and scaling slopes, using the landscape‐scale land‐use data as described in section (2.3). As detailed in the results section, since the scaling slopes were not highly responsive to the examined key environmental variables, we derived biome‐specific *c* parameters and scaling slopes to account for their variabilities in subsequent analyses by averaging the two parameters across plots within the same biome (Figure S1 and Table S2). Similarly, we derived a biome‐specific relative habitat affinity based on the corresponding diversity ratio and scaling slopes. As discussed in Note S3, a key assumption for the study is that the plot‐scale scaling slopes could be generalized to the landscape scale.

### 2.5 Estimating Fungal Diversity Loss and Gain Rates

Total fungal diversity in each pixel was estimated separately, either with the species distribution model (SDM) or the countryside SAR framework. For the former, we fit an SDM for each of the 8597 species present in ≥ 10 operational sites with three widely‐used algorithms of random forest, generalized linear model, and maximum entropy (Schmitt et al. 2017). We repeated each algorithm 10 times and used the “holdout” method for cross‐validation by splitting the occurrence data into 70% for model training and 30% for validation. Species continuous occurrence probabilities were converted to binary presence–absence data based on the thresholds that maximized the sum of sensitivity and specificity, a common practice in the SDM literature (Freeman and Moisen 2008). Finally, we assembled the outputs from the three modeling algorithms and determined the fungal diversity for each equal‐area pixel by summing the number of species predicted to be present within each pixel. For the countryside SAR framework, we estimated fungal diversity for each equal‐area pixel based on the proportion of natural and human‐dominated land areas within, biome‐specific habitat affinities, *c* parameters (i.e., baseline fungal diversity), and scaling slopes. For estimating current pixel‐scale fungal diversity with SDMs, we used mean climate data from 1970 to 2000 (WorldClim v2) and soil data from the World Soil Information Service (updated in 2023). For the countryside SAR model, we used land‐use data observed in 2015. As described in sections (2.2) and (2.3), since projections for climate and land‐use data are available for 2100, we further estimated future pixel‐scale diversity using either the SDM or countryside SAR approach, while keeping species' relative habitat affinities, *c* parameters, and scaling slopes constant over time. For both approaches, we calculated fungal diversity loss and gain rates at each pixel as the difference between current and future diversity, divided by current estimates, with negative and positive values indicating losses and gains, respectively.

### 2.6 Statistical Analyses

#### 2.6.1 Examining Current Impacts of Land‐Use Types on Soil Fungal Diversity

To test if natural land systems overall exhibited higher fungal diversity than the human‐dominated ones, we used a generalized linear mixed‐effects model to test the impact of land‐use type on the fungal gamma diversity of each human‐dominated plot and its paired natural plots. In the model, gamma diversity was included as the response variable, modeled by the Poisson distribution, while land‐use type and site identity were included as the fixed and random effects, respectively. This analysis was conducted for both the whole fungal community and individual guilds. When land‐use type significantly affected gamma diversity, the diversity ratio between the human‐dominated and natural land systems was considered significant. A diversity ratio below 1 would indicate lower diversity in the human‐dominated than in the natural land system.

#### 2.6.2 Examining Variations in the Scaling Slope Among Guilds and Environments

To compare the mean diversity scaling slope among fungal guilds, we used a one‐way ANOVA with a Welch's *t*‐test to account for unequal variance, followed by post hoc comparisons. To test the variation of the scaling slope and its leading drivers, we used a linear mixed‐effects model to examine the effect sizes of climate and soil variables and baseline fungal diversity on the scaling slope, followed by a variance partitioning analysis to quantify their relative contributions. Here, plot‐scale climate variables, soil variables, and baseline fungal diversity were treated as fixed effects, and site identity was included as a random term. For the variance partitioning analysis, only variables that significantly affected the variability of the scaling slope were selected.

#### 2.6.3 Examining the Effects of Climate and Land‐Use Change on Fungal Diversity and Their Spatial Overlap

To test if climate and land‐use changes typically exhibited a negative effect on soil fungal diversity, we first calculated the pixel‐scale diversity loss and gain rates driven by either factor, as described in section (2.5). For each biome and across biomes, we then estimated both the mean diversity loss and gain rates with a linear mixed‐effects model, as described in Note S4, along with the net diversity change rate. We then examined if the magnitude of the estimated mean loss rate typically overrode that of gains, and if both factors typically drove net diversity losses. Similarly, we estimated and compared the mean loss and gain rates among fungal guilds for each biome and across biomes. To gain further insights into the vulnerability of different biomes to climate and land‐use changes, we also compared the mean diversity loss and gain rates among biomes for both the whole community and individual guilds.

To examine the spatial overlap of climate and land‐use change effects on fungal diversity, we first determined how fungal diversity in each pixel would be affected by climate or land‐use changes (e.g., climate‐driven diversity loss) and then identified the pixels showing spatial overlap of both factors, whether driving diversity losses or gains. Finally, we determined the relative proportion of those pixels for each biome.

All data preparation, subsequent modeling and analyses were conducted in R. We mainly used the “neonMicrobe” R package for fungal data collation, the “SSDM” package for species distribution modeling, the “iNEXT”, “lme4”, and “emmeans” packages to test the effect of land‐use type on fungal community diversity, and the “terra”, “tigris”, “sp”, and “ggplot2” packages for processing spatial data and visualization (Hsieh et al. 2016; Schmitt et al. 2017).

## 3 Results

### 3.1 The Effect of Current Climate and Land‐Use Type on Soil Fungal Diversity

**FIGURE 2:**
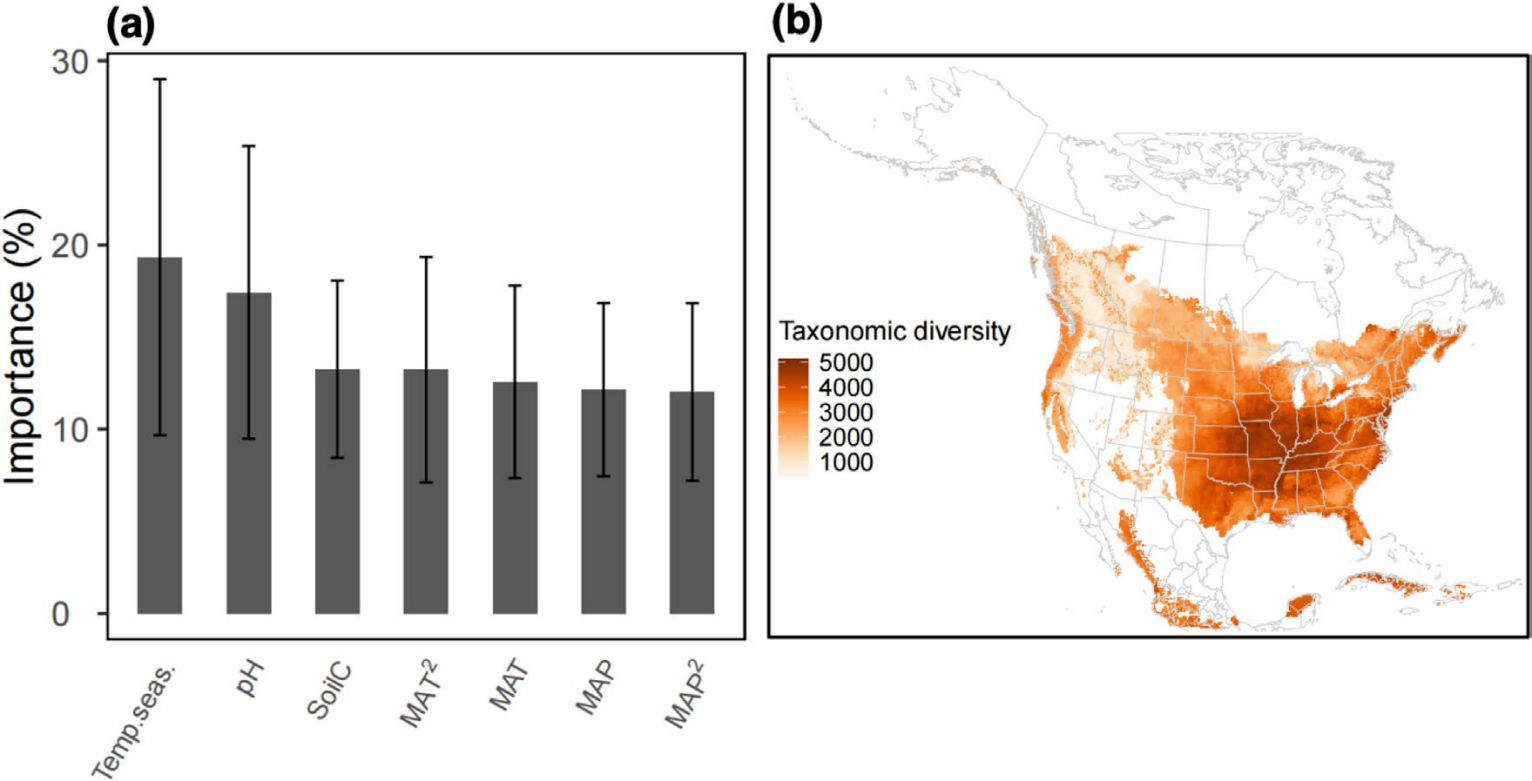
The relative importance of individual climate and soil variables in predicting species occurrences for the 8597 fungal taxa (a), and the projected current soil fungal diversity across the study region (b). The environmental variables included mean annual temperature (MAT) and its quadratic term (MAT^2^), mean annual precipitation (MAP) and its quadratic term (MAP^2^), temperature seasonality (Temp.seas.), soil pH (pH), and total soil carbon content (SoilC). Error bars in panel (a) represent the standard deviation of variable importance across species. Map lines delineate study areas and do not necessarily depict accepted national boundaries.

Temperature seasonality and mean annual temperature (MAT) were important factors in predicting soil fungal diversity across North America, both for the whole fungal community and individual guilds (Figure 2a; Figure S2). Projections stacked from the 8597 species‐level SDMs revealed fungal diversity hotspots in the midwestern and southeastern portions of North America (Figure 2b).

**FIGURE 3:**
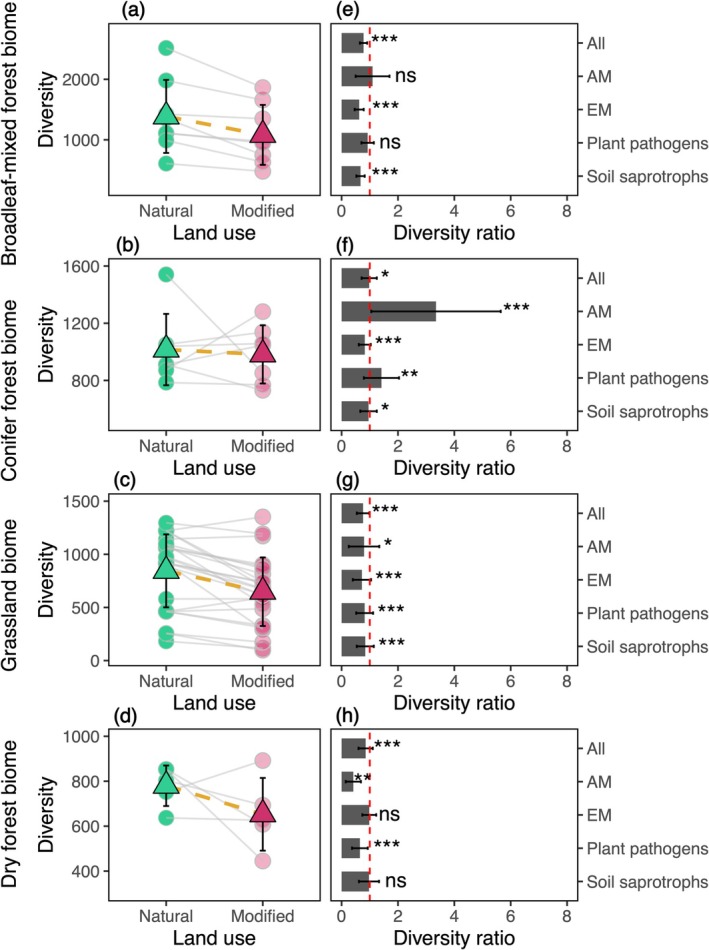
The effect of plot‐scale land‐use types on soil fungal taxonomic diversity was assessed on 45 plots in human‐dominated land systems across nine NEON sites spanning four biomes. For each biome, total fungal diversity (gamma) was compared for each human‐dominated plot and its paired adjacent natural plots with standardized sampling efforts. The gray lines in the first column panels connect diversity estimates for each of the human‐dominated plots to those of their paired natural plots, between which a diversity ratio was determined. The triangles show the mean gamma diversity across the natural and human‐dominated land systems. The second column panels display the mean diversity ratio (mean ± SD) between the human‐dominated and natural land systems for both the whole fungal community (All) and individual guilds. The dashed red line denotes a baseline value of one. For each biome, the significance of the diversity ratio was tested with a linear mixed‐effects model as described in section (2.6.1) of the main text. ***p ≤ 0.001; **p ≤ 0.01; *p ≤ 0.05; ns, nonsignificant. EM, Ectomycorrhizal mycorrhizal fungi; AM, arbuscular mycorrhizal fungi.

At the plot scale, we found strong evidence that land‐use change from natural to human‐dominated land systems typically reduced fungal diversity (Figure 3a–d). Across biomes, for the whole community, human‐dominated systems exhibited an average of 16% lower plot‐scale total fungal diversity compared to natural systems, resulting in a diversity ratio typically below one (Figure 3e–h; Table S2). However, land‐use change did not consistently reduce mycorrhizal fungal diversity while promoting that of plant pathogens, as hypothesized. While land‐use change consistently reduced ectomycorrhizal (EM) fungi and soil saprotrophs across biomes, it promoted diversity for arbuscular mycorrhizal (AM) fungi and plant pathogens in the coniferous forest biome, and decreased their diversity in the grassland biome (Figure 3f,g). Together, these findings suggest that despite some guild‐ and biome‐specific responses, soil fungi typically exhibit a lower relative affinity for human‐dominated compared to natural land systems.

### 3.2 Spatial Scaling of Fungal Diversity and Its Variations Among Guilds and Environments

**FIGURE 4:**
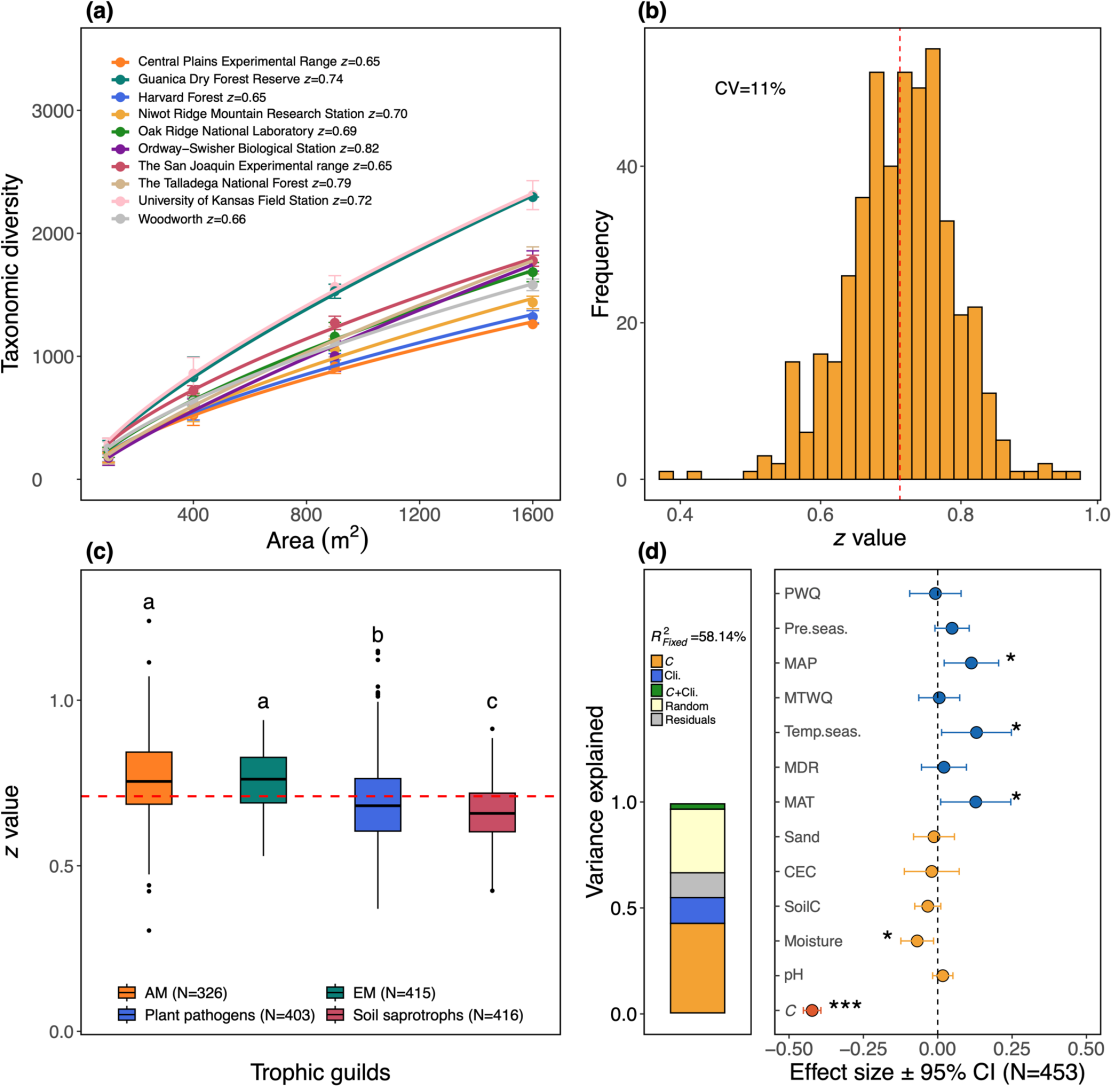
The detection, variability, and underlying drivers of the fungal species–area relationship (SAR) across 469 40 × 40 m plots. Panels (a–d) illustrate the following: (a) the plot‐scale SAR constructed from raw data for ten randomly selected plots located at different NEON sites. Each dot represents the estimated diversity of the corresponding scale (mean ± SD); (b) the histogram illustrating the distribution of the estimated scaling slopes (i.e., *z* parameters); (c) the comparison of the mean scaling slope among four fungal guilds; (d) the main drivers of the scaling slopes examined with a linear mixed‐effects model, followed by a variance partitioning analysis. The left bar plot in panel (d) shows the results of the variance partitioning among different sets of variables that exhibited significant effects on the scaling slopes, including baseline fungal diversity (i.e., *c* parameter), climatic variables (Cli.), the joint effect of baseline fungal diversity and climate (*c* + Cli.), random effects (Random), and residuals. Each fraction represents the contribution of each set of variables to the variance explained by the fixed effects of the model. The right forest plot in panel (d) displays the effect size of individual variables on the scaling slopes derived from the mixed‐effects model that included 13 variables: Precipitation of the warmest quarter (PWQ), precipitation seasonality (Pre.seas.), temperature seasonality (Temp.seas.), mean annual precipitation (MAP), mean annual temperature (MAT), mean temperature of the wettest quarter (MTWQ), mean diurnal range (mean of monthly (max temperature‐min temperature), MDR), soil sand content (Sand), cation exchange capacity (CEC), soil carbon content (SoilC), soil moisture, pH, and baseline fungal diversity (i.e., *c* parameter). ***p ≤ 0.001; *p ≤ 0.05.

Given the observed effects of land‐use change on soil fungal diversity, we next examined how fungal diversity scaled with habitat area, a potential way to estimate landscape‐scale fungal diversity. At the plot level, we demonstrated widespread SAR scaling relationships between soil fungal diversity and habitat area (Figure 4a). Across 469 plots, the estimated scaling slopes (i.e., *z* parameters) ranged from 0.37 to 0.77 with a mean of 0.71 and a coefficient of variation of 11% (Figure 4b). Mycorrhizal fungi, especially AM fungi, displayed the steepest mean scaling slope, followed by plant pathogens and soil saprotrophs (Figure 4c). These findings suggest that soil fungal communities are strongly spatially structured rather than uniformly distributed. Further, the rate at which fungal diversity scales with habitat area could depend on species' life‐history strategies, such as dispersal capacity and host plant dependency. Importantly, the observed diversity scaling slopes laid the foundation for estimating landscape‐scale fungal diversity.

Since the scaling slopes varied in different comparisons, we next tested how this variation could be explained by key climate and soil variables and baseline fungal diversity (i.e., *c* parameters) with a linear mixed‐effects model, aiming to assess the extent of context dependency in scaling slopes across regions. We found that regions with higher MAT, mean annual precipitation, and temperature seasonality were associated with steeper scaling slopes, while regions with higher soil moisture and baseline fungal diversity corresponded with flatter slopes (Figure 4d). The contrasting effects of MAP and soil moisture may reflect the presence of woody wetlands in some high‐MAP regions, where persistently waterlogged soils favor a narrow set of moisture‐tolerant fungi, resulting in reduced community variability. Of the 58% of variability in the scaling slopes explained by the model, 42% was uniquely attributed to baseline fungal diversity, 12% to climate variables, and approximately 1% to soil variables (Figure 4d). These patterns were broadly observed for guild‐level scaling slopes (Figure S3), suggesting that climate and baseline fungal diversity are the primary drivers of variations in scaling slopes. Given that biomes broadly reflect regional climate and diversity patterns, we derived biome‐ and guild‐specific scaling slopes and *c* parameters to quantify the landscape‐scale fungal diversity in subsequent analyses. Together with the identified key climate variables shaping current fungal distributions, we then quantified climate‐ and land‐use‐driven fungal diversity changes by modeling changes in climate suitability or effective land area over time across landscapes.

### 3.3 Potentially Distinct Effects of Climate and Land‐Use Changes on Soil Fungal Diversity

**FIGURE 5:**
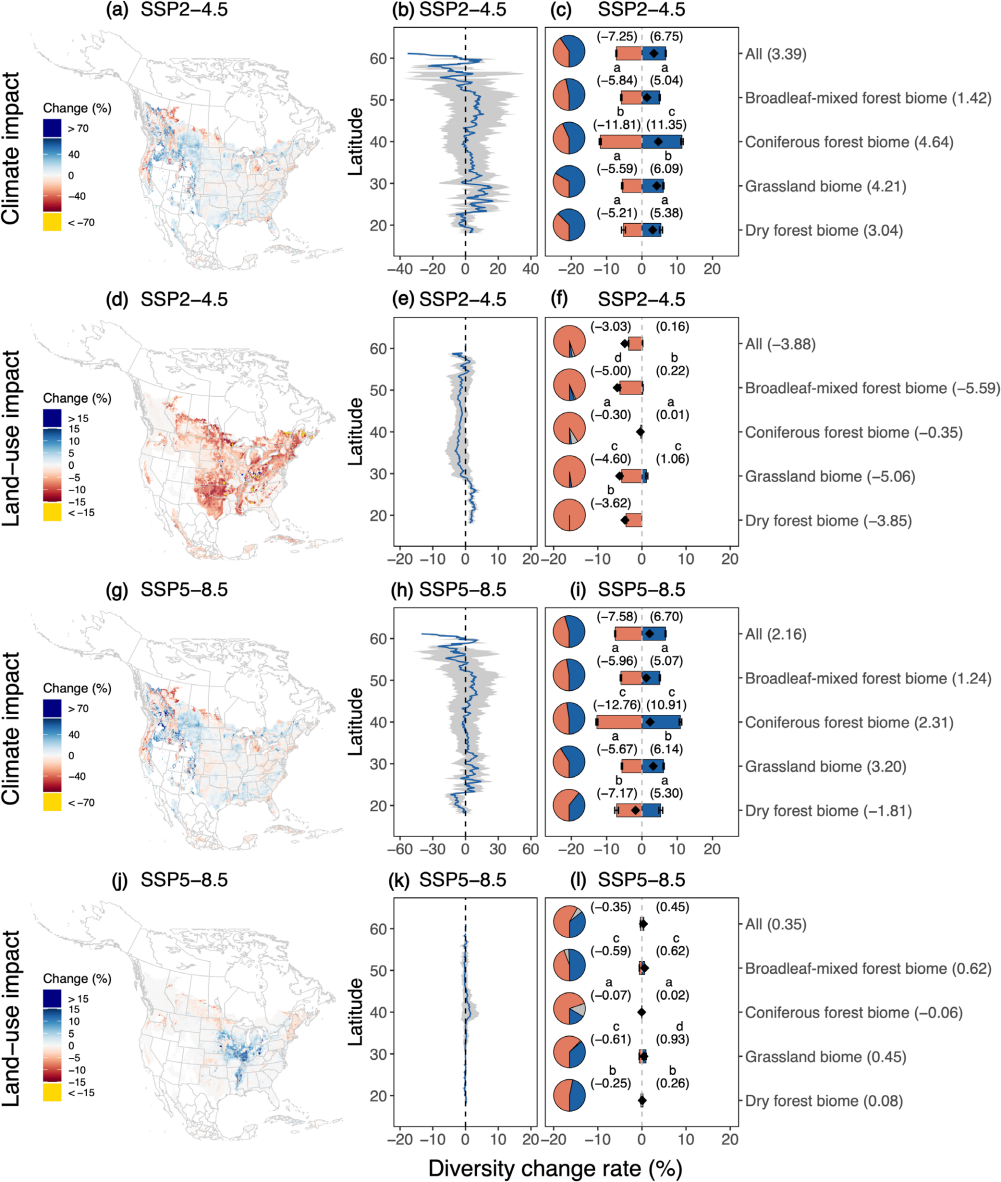
The effects of climate (a–c and g–i) and land‐use changes (d–f and j–l) on whole fungal community diversity losses and gains under moderate‐ (SSP2–4.5) and high‐emission scenarios (SSP5–8.5). Panel (a) shows the spatial variation of climate‐driven diversity loss and gain rates; panel (b) shows the net climate‐driven diversity change rate along latitudes (mean ± SE). For panel (c), the pie charts show the proportion of pixels predicted to undergo diversity losses (coral), gains (blue), or no changes (grey). The coral and blue bars indicate the model‐estimated mean diversity loss and gain rates driven by climate (mean ± 95% CI), respectively. The black diamonds, with their values displayed on the right y‐axis, indicate the net diversity change rate determined with the raw data. Mean diversity loss and gain rates were estimated for each biome of temperate broadleaf and mixed forests (broadleaf‐mixed forest biome), temperate coniferous forests (coniferous forest biome), temperate grasslands (grassland biome), savannas and shrublands, and tropical and subtropical dry broadleaf forests (dry forest biome), and across four biomes (All). Panels (d–f) and (j–l) show the effects of land‐use change on soil fungal diversity. The letters on the bars represent differences in the mean diversity loss and gain rates among biomes. Bars that share a letter do not differ significantly in their means. For the cross‐biome diversity loss and gain rates, only the estimated means are shown, without reference to specific biomes. Map lines delineate study areas and do not necessarily depict accepted national boundaries.

Partially supporting our hypothesis, our model predicted that climate change typically drove both diversity losses and gains, with effects being stronger under SSP5–8.5. Land‐use change predominantly drove diversity losses under SSP2–4.5, with diminished effects under SSP5–8.5. Specifically, the climate‐driven diversity change rate ranged from −72% to 150% across biomes under SSP2–4.5, with higher means predicted in the coniferous forest biome (mean ± CI: −11.8% ± 0.3% for losses, 11.4% ± 0.4% for gains; Figure 5a–c). Within biomes, diversity losses and gains were generally comparable in magnitude, but gains occurred more frequently, resulting in net gains across biomes (mean ± SD: 3.4% ± 15.9%; Figure 5c; Figure S4a–d). In comparison, the land‐use‐driven diversity change rate ranged from −22.1% to 15.0% across biomes, with greater losses in broadleaf‐mixed forest (mean ± CI: −5.0% ± 0.1%) and grassland biomes (mean ± CI: −4.6% ± 0.1%; Figure 5f), leading to net losses across biomes (mean ± SD: −3.9% ± 4.1%). Transitioning from SSP2–4.5 to SSP5–8.5, climate‐driven diversity changes intensified with reduced net gains, while land‐use‐driven diversity changes shifted from net losses to minimal gains (Figure 5f,l).

Contrary to our initial hypothesis, neither climate nor land‐use change predominantly drove diversity losses in mycorrhizal fungi while promoting gains in plant pathogens. Climate‐driven diversity loss rates were highest for AM fungi and lowest for soil saprotrophs. Similarly, diversity gain rates were highest for AM fungi and lowest for EM fungi (Figure S5a,c). In contrast, land‐use change typically caused high diversity loss rates for EM fungi without significantly promoting gains in plant pathogens (Figure S5b,d).

### 3.4 The Spatial Overlap of Climate and Land‐Use Change Effects on Soil Fungal Diversity

**FIGURE 6:**
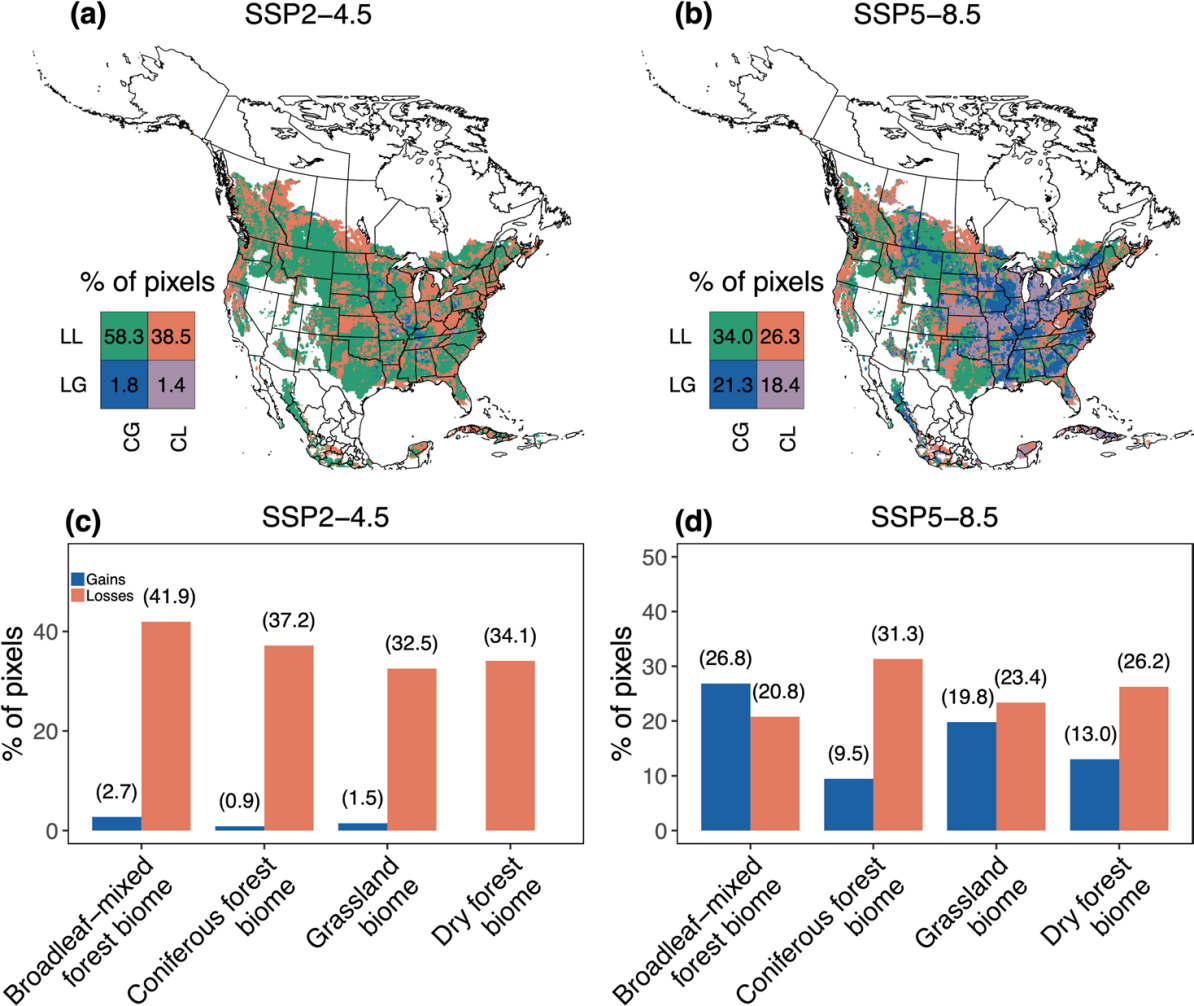
The bivariate map showing the spatial overlap of climate and land‐use change effects on the whole‐community fungal diversity change under moderate‐ (SSP2–4.5) and high‐emission scenarios (SSP5–8.5). Pixels in panels (a) and (b) are colored to represent various combinations of climate and land‐use change effects, which are classified as either causing diversity losses due to climate change (CL) or land‐use change (LL), or diversity gains due to climate change (CG) or land‐use change (LG). The numbers in the legend represent the proportion of pixels with various combinations of climate and land‐use change effects. Panels (c) and (d) illustrate the proportion of pixels in each biome where both factors were predicted to drive diversity losses or gains. Map lines delineate study areas and do not necessarily depict accepted national boundaries.

Our hypothesis that both climate and land‐use change would mainly drive fungal diversity change in the broadleaf‐mixed forest biome was partially supported. Across emission scenarios, for the whole fungal community, EM fungi, and soil saprotrophs, both factors were predicted to drive diversity losses across a broad north–south swath of the temperate grassland biome, Pacific Northwest coniferous forest biome, and Appalachian Mountains region, with a higher spatial overlap occurring in the coniferous forest biome (> 30%; Figure 6; Figures S6c–f and S7c–f). For diversity gains, the overlap was largely predicted under SSP5–8.5, driven by expanded land‐use‐driven diversity gains, with a greater proportion (> 22%) occurring in the broadleaf‐mixed forest biome (Figure 5j). For AM fungi and plant pathogens, both factors were predicted to drive diversity losses across a broader extent of the grassland biome (> 22%), whereas diversity gains were largely predicted in the coniferous forest biome (> 35%; Figure S7a,b,g,h).

## 4 Discussion

Our model suggests that, following the historical patterns of socioeconomic development outlined in the SSP2–4.5 scenarios, climate change would typically drive both fungal diversity losses and gains, especially in northwestern coniferous forest biomes and among AM fungi, potentially reshuffling landscape‐scale fungal community composition. In contrast, land‐use change would predominantly drive diversity losses, particularly in broadleaf‐mixed forest and grassland biomes, and among EM fungi, potentially imposing additional barriers to fungal establishment beyond those induced by climate unsuitability. While adopting the SSP5–8.5 scenarios might mitigate land‐use effects, it would intensify climate‐driven fungal diversity changes. Crucially, both climate and land‐use changes were predicted to drive whole‐community fungal diversity losses across over one‐third of coniferous forest biomes regardless of emission scenarios. These findings highlight the need for biome‐ and guild‐specific conservation strategies to mitigate the potentially adverse and spatially heterogeneous effects of climate and land‐use changes on soil fungal diversity.

### 4.1 The Effect of Current and Future Climate on Soil Fungal Diversity

Our study reinforces the importance of key climate factors such as temperature and precipitation in driving broad‐scale fungal diversity patterns (Větrovský et al. 2019; Qin et al. 2023). These factors were similarly important in shaping guild‐specific fungal distributions, suggesting that broad fungal groups share ecological limitations imposed by regional thermal conditions (Qin et al. 2023). The higher diversity loss and gain rates typically projected in the northwestern coniferous forest biome align with the idea that warming‐induced drought would intensify in these habitats, where fungal communities occupy vulnerable climate niches, potentially accelerating species redistributions (Tepley et al. 2017; Qin et al. 2023). Across biomes, climate change, broadly associated with warming and increased precipitation, tended to drive net diversity gains, partially consistent with the globally positive effect of warming on fungal diversity, likely driven by increased energy availability (Zhou et al. 2020). However, as hypothesized, both the frequency and magnitude of climate‐driven diversity losses increased under SSP5–8.5, implying that intensified warming may restrict species' distributions either by exceeding species' thermal limits or by decreasing their competitiveness (Větrovský et al. 2019) (Figures 5c,i and 6a,b). Notably, the predicted climate‐driven diversity losses and gains suggest potential shifts in spatial location rather than losses of fungal climate niches (Steidinger et al. 2020; Van Nuland et al. 2024).

Beyond examining general diversity change rates for broad fungal guilds and whole‐fungal communities, our study demonstrates how climate change may alter the distribution of key plant pathogens (Figure S8). By focusing on four major pathogenic fungal genera, our predictions suggest that both the diversity (i.e., mean richness per grid) and the geographic range of *Venturia* spp., *Alternaria* spp., and *Fusarium* spp. are likely to increase in temperate broad‐leaved forest biomes under the SSP5–8.5 scenario. Conversely, these metrics were projected to increase in grassland biomes for *Botrytis* spp. Given their potentially negative impacts on plant fitness—such as root rot caused by *Fusarium* species, apple scab caused by *Venturia inaequalis*, and grey mold caused by *Botrytis cinerea*—these projections raise concerns about increased pest pressure in both natural forest ecosystems and plantations (Williamson et al. 2007; Holeski et al. 2009; Rocafort et al. 2022). Importantly, these projections are largely based on the climatic niche suitability for fungi and do not account for interactions among host susceptibility, climate conditions, and forest management practices, all of which could significantly influence disease severity. Future research targeting these points, alongside continued efforts in disease monitoring, forecasting, planning, and mitigation strategies, will be essential for developing a comprehensive understanding and informing effective responses to these potential impacts (Sturrock et al. 2011).

### 4.2 The Effect of Current and Future Land‐Use Scenarios on Soil Fungal Diversity

In comparison, land‐use impacts on fungal diversity depended more on trophic guilds and biomes. Under SSP2–4.5, land‐use change was predicted to predominantly drive whole‐community fungal diversity losses, especially in broadleaf‐mixed forest and grassland biomes (Figure 5d−f). These patterns were likely driven by the continued biofuel cropland expansion aimed at partially transitioning away from fossil fuels (Riahi et al. 2017). Among fungal guilds, EM fungi typically exhibited the greatest diversity losses, reflecting their high sensitivity to deforestation that disrupts host‐specific symbiosis (Qu et al. 2024). In contrast, AM fungi and plant pathogens were predicted to gain diversity in the coniferous forest biome, where human‐dominated land systems tended to support higher diversity for these groups compared to natural ones (Figure 3f). These patterns may be driven by the conversion of EM tree‐dominated vegetation to grass‐dominated ones, which are more reliant on AM fungi and more susceptible to pathogens due to reduced plant defenses (Roossinck and García‐Arenal 2015; Labouyrie et al. 2023). The predicted diversity gains, albeit weak, driven by land‐use changes under SSP5–8.5 (Figure 5f,l), may result from slight increases in natural land systems due to responsible land‐use practices and habitat protection supported by increased social capital (Pang et al. 2024). Combined, these findings suggest that, compared to climate effects, land‐use change may exert more guild‐specific impacts and may shift the dominant fungal guilds in a landscape by altering host plant communities (Qu et al. 2024).

### 4.3 Spatial Overlap of Climate and Land‐Use Effects on Soil Fungal Diversity

Despite their potentially distinct effects on soil fungal diversity, both climate and land‐use changes were predicted to drive diversity losses with greater spatial overlap in temperate coniferous forest biomes (for the whole fungal community, EM fungi, and soil saprotrophs) or in grassland biomes (for AM fungi and plant pathogens). These patterns suggest that fungal communities in these regions are likely to face both unsuitable climates and degraded habitats, warranting prioritized conservation efforts. However, in applying the countryside SAR framework, land‐use effects were largely estimated through changes in landscape‐scale effective land area. While the projected land‐use data may be driven by the novel climate under certain emission scenarios, the challenges of incorporating climate variables into the countryside SAR framework limit the quantification of their potentially interactive effects (Zhou et al. 2024). Assuming synergistic interactions, we expect that regions where both factors were predicted to drive fungal diversity losses might experience greater losses than would be expected from either factor alone, as land‐use change often creates homogeneous landscapes with reduced climate buffering capacity (Ordonez et al. 2014). For regions where climate and land‐use changes exhibited opposing effects, the net impact on fungal diversity remains uncertain. Assuming simply additive effects, the resulting patterns of whole‐community fungal diversity change resembled those driven by climate alone, being greater in coniferous forest biomes (Figures S9 and S10). Despite these uncertainties, the projections offer valuable insights into how incorporating the spatial heterogeneity of multiple global changes could enhance conservation efficiency (Tedersoo et al. 2022). For example, in regions predicted to mainly undergo climate‐driven diversity losses (e.g., upper Midwest), adaptation strategies like identifying fungi climate refugia and preserving fungal spores may increase ecosystem resilience and facilitate future restoration (Willing et al. 2024). Conversely, regions facing primarily land‐use‐driven diversity losses (e.g., southern grassland biome) may benefit from management strategies like land acquisition to protect remaining habitats and support species adaptation (Lark et al. 2020). Future studies that integrate both climate and land‐use variables into a single SDM could offer a more robust approach to quantify their individual and interactive effects on both fungal community diversity and composition.

### 4.4 The Spatial Scaling of Fungal Diversity and Its Variations Among Guilds and Environments

Toward our overarching goal of quantifying landscape‐scale fungal diversity, this study also revealed how species life‐history strategies and climate could shape spatial scaling patterns of fungal diversity. The steeper scaling slopes observed for mycorrhizal fungi may reflect the intense interspecific competition for host roots and carbohydrates, increasing community monopolization and spatial turnover (Engelmoer et al. 2014). By contrast, the flatter slopes observed for soil saprotrophs likely reflect their broader substrate niches and greater dispersal capacities, homogenizing community compositions over space (Baldrian et al. 2022). Based on 469 SARs, our study further suggests that soil fungi may inherently exhibit steeper scaling slopes than previously proposed (Green and Bohannan 2006). If so, even small losses of original habitat could cause substantial fungal diversity declines (Drakare et al. 2006). Likewise, variations in scaling slopes over environments imply that land‐use‐driven diversity declines may be more pronounced in warmer regions or areas with higher temperature seasonality (e.g., Midwestern and southern United States). However, our study also suggests that land‐use change may create novel habitats for certain fungal guilds, with its effects likely varying by management practices and biomes (Qu et al. 2024). Although this study compared fungal diversity between human‐dominated and natural systems using thousands of soil samples and accounted for diverse agricultural practices such as crop rotation, prescribed burning, and grazing, the spatial coverage of human‐dominated plots was still limited. Consequently, the estimated effects of land‐use change on fungal diversity relied on the key assumption that conversions from natural to human‐dominated systems have broadly comparable impacts within the same biome, given their shared climate, soil conditions, and dominant vegetation (Olson et al. 2001). This limitation cautions against making broad generalizations to other systems without further verification and underscores the need for expanded and standardized sampling to more accurately assess landscape‐scale land‐use impacts on soil fungal diversity (Balami et al. 2020).

To conclude, our study suggests that under the SSP2–4.5 scenario, climate change is likely to reshuffle landscape‐scale fungal communities by driving both diversity losses and gains, particularly in the coniferous forest biome and among AM fungi. In contrast, land‐use change would predominantly drive fungal diversity losses, especially in broadleaf‐mixed forest biomes and among EM fungi. While increasing reliance on fossil fuels, as represented by the SSP5–8.5 scenarios, may mitigate the negative effects of land‐use change, it would exacerbate climate‐driven fungal diversity losses. Given that both climate and land‐use changes were predicted to drive diversity losses, regions such as the northwestern coniferous forest and southern grassland biomes may serve as important soil fungal conservation hotspots. Our study underscores the need for biome‐ and guild‐specific conservation strategies and the development of more sustainable socioeconomic development pathways to safeguard soil fungi and the critical ecosystem functions they support.

## Author Contributions

**Wenqi Luo:** conceptualization, formal analysis, methodology, visualization, writing – original draft, writing – review and editing; **Kabir G. Peay:** conceptualization, data curation, writing – review and editing; **Thiago Gonçalves‐Souza:** methodology, writing – review and editing; **Peter B. Reich:** conceptualization, methodology, funding acquisition, supervision, writing – review and editing; **Donald R. Zak:** conceptualization, supervision, writing – review and editing; **Kai Zhu:** conceptualization, data curation, funding acquisition, supervision, validation, writing – original draft, writing – review and editing.

## Conflicts of Interest

The authors declare no conflicts of interest.

## Supporting information


**Table S1:** Details on the location, historical and current land‐use types, and current main disturbance types of the 45 human‐dominated plots.
**Table S2:** The estimated biome‐ and guild‐specific parameters for the countryside SAR framework.
**Figure S1:** The distribution of the studied 128 operational sites among 15 biomes across North America.
**Figure S2:** The importance of individual environmental variables in predicting guild‐level fungal species occurrence.
**Figure S3:** The underlying drives of the guild‐level scaling slopes.
**Figure S4:** Comparing the guild‐level mean diversity loss and gain rates among biomes with diversity losses and gains driven either by climate or land‐use change under both moderate‐ (SSP2–4.5) and high‐emission scenarios (SSP5–8.5).
**Figure S5:** Comparison of the mean diversity loss and gain rates among fungal guilds with diversity losses and gains driven either by climate or land‐use changes under both moderate‐ (SSP2–4.5) and high‐emission scenarios (SSP5–8.5).
**Figure S6:** The bivariate map showing the spatial overlap of climate and land‐use change effects on the guild‐level fungal diversity losses and gains under moderate‐ (SSP2–4.5) and high‐emission scenarios (SSP5–8.5).
**Figure S7:** The spatial overlap of climate and land‐use change effects on the guild‐specific fungal diversity losses and gains in each biome under moderate‐ (SSP2–4.5) and high‐emission scenarios (SSP5–8.5).
**Figure S8:** Current and projected diversity (i.e., mean richness per grid cell) and occupancy for four common plant pathogenic fungal genera.
**Figure S9:** The potentially combined effect of climate and land‐use changes on soil fungal diversity change rate for the whole fungal communities under moderate‐ (SSP2–4.5) and high‐emission scenarios (SSP5–8.5).
**Figure S10:** The predicted spatial extent of diversity losses and gains driven by the potentially combined effect of climate and land‐use changes under moderate‐ (SSP2–4.5) and high‐emission scenarios (SSP5–8.5).
**Figure S11:** A schematic diagram showing the construction of a plot‐scale SAR.
**Figure S12:** The determination of a site‐scale SAR and the relationship between scaling slopes at plot and landscape scales.
**Figure S13:** Comparison of the distance‐decay pattern of soil fungal communities examined at the 40 × 40 m plot and 10 × 10 km landscape scales.
**Note S1**. Determination of the historical land‐use type for NEON plots.
**Note S2**. Derivation of habitat affinity for the countryside SAR.
**Note S3**. Testing the generalization of plot‐scale scaling slopes to the landscape scale.
**Note S4**. Testing the difference in mean diversity loss and gain rates among biomes and fungal guilds.

## Data Availability

The data that support the findings of this study include openly available fungal data from the National Ecological Observatory Network at https://doi‐org.proxy.lib.umich.edu/10.48443/ybrs‐zv89, RELEASE‐2021 (DP1.10086.001). The data from the Dimensions of Ectomycorrhizal Biodiversity project are available from the NCBI Sequence Read Archive via accession number PRJNA950128. Historical and future land‐use data are available at https://doi‐org.proxy.lib.umich.edu/10.6084/m9.figshare.12967760 and https://doi.org/10.5281/zenodo.6469247. Intermediate data products and reproducible R code are available on GitHub at https://github.com/zhulabgroup/soil‐fungi‐sar/tree/main.
