# Supplementary material for: Climate and Land‐Use Changes Predicted to Jointly Drive Soil Fungal Diversity Losses in One‐Third of North American Coniferous Forests
Source: Glob Chang Biol. 2025 Nov 8;31(11):e70598. doi: 10.1111/gcb.70598 (PMC12595595; doi:10.1111/gcb.70598)
Supplement: Supplementary file 1 — Table S1: Details on the location, historical and current land‐use types, and current main disturbance types of the 45 human‐dominated plots. Table S2: The estimated biome‐ and guild‐specific parameters for the countryside SAR framework. Figure S1: The distribution of the studied 128 operational sites among 15 biomes across North America. Figure S2: The importance of individual environmental variables in predicting guild‐level fungal species occurrence. Figure S3: The underlying drives of the guild‐level scaling slopes. Figure S4: Comparing the guild‐level mean diversity loss and gain rates among biomes with diversity losses and gains driven either by climate or land‐use change under both moderate‐ (SSP2–4.5) and high‐emission scenarios (SSP5–8.5). Figure S5: Comparison of the mean diversity loss and gain rates among fungal guilds with diversity losses and gains driven either by climate or land‐use changes under both moderate‐ (SSP2–4.5) and high‐emission scenarios (SSP5–8.5). Figure S6: The bivariate map showing the spatial overlap of climate and land‐use change effects on the guild‐level fungal diversity losses and gains under moderate‐ (SSP2–4.5) and high‐emission scenarios (SSP5–8.5). Figure S7: The spatial overlap of climate and land‐use change effects on the guild‐specific fungal diversity losses and gains in each biome under moderate‐ (SSP2–4.5) and high‐emission scenarios (SSP5–8.5). Figure S8: Current and projected diversity (i.e., mean richness per grid cell) and occupancy for four common plant pathogenic fungal genera. Figure S9: The potentially combined effect of climate and land‐use changes on soil fungal diversity change rate for the whole fungal communities under moderate‐ (SSP2–4.5) and high‐emission scenarios (SSP5–8.5). Figure S10: The predicted spatial extent of diversity losses and gains driven by the potentially combined effect of climate and land‐use changes under moderate‐ (SSP2–4.5) and high‐emission scenarios (SSP5–8.5). Figure S11: A schema [file GCB-31-e70598-s001.pdf]

Supplementary Information

Climate and land-use changes predicted to jointly drive soil fungal diversity losses in one-third of North American coniferous forests

Wenqi Luo[1*], Kabir Peay[2, 3], Thiago Gonçalves-Souza[1, 4], Peter B. Reich[1, 5], Donald R Zak[1], Kai Zhu[1*]

1. Institute for Global Change Biology and School for Environment and Sustainability, University of Michigan, Ann Arbor, MI, USA
2. Department of Biology, Stanford University, Stanford, CA, USA
3. Department of Earth System Science, Stanford University, Stanford, CA, USA
4. Department of Ecology and Evolutionary Biology, University of Michigan, Ann Arbor, MI, USA
5. Department of Forest Resources, University of Minnesota, St. Paul, MN, USA

* Author for correspondence:
Wenqi Luo (wenqil@umich.edu)
Kai Zhu (zhukai@umich.edu)

Table S1 Details on the location, historical and current land-use types, and current main disturbance types of the 45 human-dominated plots. Information on the main disturbance types for each plot was inferred from NEON site descriptions (https://www.neonscience.org/field-sites). The contrasted land-use type refers to the land-use categories selected for the natural plots when comparing fungal diversity between a human-dominated plot and its adjacent natural plots. For example, for the first human-dominated plot (BLAN_033), its historical land-use type was forest, but it has since been converted to cultivated cropland. To examine the effect of land-use change from forest to cropland on fungal diversity, we compared fungal diversity in the cultivated cropland plot to that in several adjacent natural plots that have remained forested over time. In this specific case, the land-use types for the selected contrasted natural plots could be evergreen forest and deciduous forest, as the historical land-use type for the cultivated cropland plot was broadly defined as forest. The disturbance types give information on the site-level landscape characteristics and land management practices. ① (An agriculture site on privately owned land. Various crops are grown across the site, including winter wheat, millet, and maize. The primary crop is winter wheat grown in a wheat-fallow rotation); ② (A few areas are cleared for pasture, hay, or other agricultural or general use); ③ (Open to the public for recreational opportunities. A few areas are cleared by burning for idle pasture, hay, or other agriculture or general uses); ④ (Cropland covers a large portion of the landscape. Supports a wide array of research and educational programs such as land use and invasive species); ⑤ (Over 85% of the sampling area is cultivated crops. The primary crop is winter wheat grown in a wheat-fallow rotation. Other crops include millet, maize, sorghum, triticale, and sunflower. No-till cropping system. Non-irrigated cropland, with a few areas of pastureland); ⑥ (Crops vary yearly (e.g., wheat, corn, milo, soybeans, alfalfa, and oats). It's possible to see the dairy cows from some plots. Research activities are related to cropping and tilling systems, management practices, and weed science); ⑦ (Prescribed burns at a frequency of 3-4 years. Removal of non-native invasive plants and grasses as well as the removal of agricultural ditches); ⑧ (Prescribed fire); ⑨ (Crop types are rotated depending on current research priorities. Research activities are related to the production efficiency of new and traditional crops).

Biomes	PlotID	Historical land-use type	Current land-use type	Contrasted land-use type	Disturbance types
Broadleaf-mixed forest[1]	BLAN_033	Forest	Cultivated crops	Evergreen forest, Deciduous forest	①
Broadleaf-mixed forest	BLAN_031	Forest	Cultivated crops	Evergreen forest, Deciduous forest	①
Broadleaf-mixed forest	BLAN_003	Forest	Pasture/Hay	Evergreen forest, Deciduous forest	①
Broadleaf-mixed forest	BLAN_038	Forest	Cultivated crops	Evergreen forest, Deciduous forest	①
Broadleaf-mixed forest	BLAN_004	Forest	Pasture/Hay	Evergreen forest, Deciduous forest	①
Broadleaf-mixed forest	SCBI_006	Forest	Pasture/Hay	Deciduous forest, Evergreen forest	②
Broadleaf-mixed forest	ORNL_021	Forest	Pasture/Hay	Deciduous forest, Evergreen forest	③
Broadleaf-mixed forest	SERC_007	Grassland	Cultivated crops	Deciduous forest	④
Grassland biome	STER_031	Grassland	Cultivated crops	Grassland herbaceous	②
Grassland biome	STER_029	Grassland	Cultivated crops	Grassland herbaceous	⑤
Grassland biome	STER_005	Grassland	Cultivated crops	Grassland herbaceous	⑤
Grassland biome	STER_026	Grassland	Cultivated crops	Grassland herbaceous	⑤
Grassland biome	STER_027	Grassland	Cultivated crops	Grassland herbaceous	⑤
Grassland biome	STER_018	Grassland	Cultivated crops	Grassland herbaceous	⑤
Grassland biome	STER_006	Grassland	Cultivated crops	Grassland herbaceous	⑤
Grassland biome	STER_011	Grassland	Cultivated crops	Grassland herbaceous	⑤
Grassland biome	STER_012	Grassland	Cultivated crops	Grassland herbaceous	⑤
Grassland biome	STER_010	Grassland	Cultivated crops	Grassland herbaceous	⑤
Grassland biome	STER_016	Grassland	Cultivated crops	Grassland herbaceous	⑤
Grassland biome	STER_032	Grassland	Cultivated crops	Grassland herbaceous	⑤

Grassland biome	STER_028	Grassland	Cultivated crops	Grassland herbaceous	⑤
Grassland biome	STER_033	Grassland	Cultivated crops	Grassland herbaceous	⑤
Grassland biome	STER_035	Grassland	Cultivated crops	Grassland herbaceous	⑤
Grassland biome	STER_034	Grassland	Cultivated crops	Grassland herbaceous	⑤
Grassland biome	KONA_003	Grassland	Cultivated crops	Grassland herbaceous	⑥
Grassland biome	KONA_001	Grassland	Cultivated crops	Grassland herbaceous	⑥
Grassland biome	KONA_005	Grassland	Cultivated crops	Grassland herbaceous	⑥
Grassland biome	KONA_006	Grassland	Cultivated crops	Grassland herbaceous	⑥
Grassland biome	KONA_004	Grassland	Cultivated crops	Grassland herbaceous	⑥
Grassland biome	KONA_002	Grassland	Cultivated crops	Grassland herbaceous	⑥
Grassland biome	KONA_059	Forest	Cultivated crops	Deciduous forest	⑥
Grassland biome	KONA_048	Forest	Cultivated crops	Deciduous forest	⑥
Grassland biome	KONA_054	Forest	Cultivated crops	Deciduous forest	⑥
Coniferous forest biome	DSNY_041	Wetland	Pasture/Hay	Woody wetlands	⑦
Coniferous forest biome	DSNY_009	Wetland	Pasture/Hay	Woody wetlands	⑦
Coniferous forest biome	DSNY_016	Forest	Pasture/Hay	Evergreen forest, Deciduous forest	⑦
Coniferous forest biome	DSNY_043	Wetland	Pasture/Hay	Woody wetlands	⑦
Coniferous forest biome	DSNY_042	Wetland	Pasture/Hay	Woody wetlands	⑦
Coniferous forest biome	DSNY_044	Wetland	Pasture/Hay	Woody wetlands	⑦
Coniferous forest biome	JERC_004	Forest	Cultivated crops	Evergreen forest, Mixed forest, Deciduous forest	⑧
Dry forest biome	LAJA_044	Forest	Pasture/Hay	Evergreen forest	⑨

Dry forest biome	LAJA_042	Forest	Pasture/Hay	Evergreen forest	⑨
Dry forest biome	LAJA_046	Forest	Pasture/Hay	Evergreen forest	⑨
Dry forest biome	LAJA_051	Forest	Pasture/Hay	Evergreen forest	⑨
Dry forest biome	LAJA_002	Forest	Cultivated crops	Evergreen forest	⑨

[1]Broadleaf-mixed forest biome

Table S2 The estimated biome- and guild-specific parameters for the countryside SAR framework for both the whole fungal communities (All) and four individual guilds. The four biomes included temperate broadleaf and mixed forests (Broadleaf-mixed forest biome), temperate coniferous forests (Coniferous forest biome), temperate grasslands (Grassland biome), and savannas and shrublands, and tropical and subtropical dry broadleaf forests (Dry forest biome). For each biome, the diversity ratio was quantified as the mean fungal gamma diversity ratio between the human-dominated and their paired natural plots, with the standard deviation also reported. c and z correspond to the parameters of the SAR model: $S = cA^z$, where S is the fungal taxonomic diversity, A is the area, c is a constant indicating local fungal diversity estimations, and z denotes the rate at which fungal taxonomic diversity increases with the sampling area (e.g., scaling slope). Species relative affinity for the natural land system was set to one, while for the the human-dominated land system, it was quantified as $affinity = (diversity\ ratio)^{\frac{1}{z}}$. EM, Ectomycorrhizal mycorrhizal fungi; AM, arbuscular mycorrhizal (AM) fungi.

Biome	Guild	Diversity ratio	sd	c	z	Affinity
Broadleaf-mixed forest biome	All	0.78	0.13	6.24	0.73	0.71
Broadleaf-mixed forest biome	AM	1.10	0.60	0.04	0.78	1.13
Broadleaf-mixed forest biome	EM	0.62	0.16	0.60	0.77	0.54
Broadleaf-mixed forest biome	Plant pathogens	0.92	0.22	0.24	0.72	0.90
Broadleaf-mixed forest biome	Soil saprotrophs	0.67	0.15	1.94	0.66	0.55
Coniferous forest biome	All	0.98	0.27	6.59	0.72	0.97
Coniferous forest biome	AM	3.35	2.30	0.07	0.79	4.63
Coniferous forest biome	EM	0.82	0.21	0.64	0.75	0.77
Coniferous forest biome	Plant pathogens	1.41	0.62	0.23	0.73	1.61
Coniferous forest biome	Soil saprotrophs	0.96	0.29	2.09	0.63	0.93
Grassland biome	All	0.76	0.22	8.60	0.72	0.69

Grassland biome	AM	0.80	0.54	0.13	0.79	0.75
Grassland biome	EM	0.72	0.32	0.38	0.80	0.66
Grassland biome	Plant pathogens	0.82	0.30	0.63	0.65	0.73
Grassland biome	Soil saprotrophs	0.84	0.30	1.49	0.68	0.77
Dry forest biome	All	0.85	0.25	9.05	0.71	0.80
Dry forest biome	AM	0.42	0.27	0.05	0.77	0.32
Dry forest biome	EM	0.98	0.25	0.56	0.76	0.98
Dry forest biome	Plant pathogens	0.65	0.28	0.41	0.68	0.53
Dry forest biome	Soil saprotrophs	0.97	0.36	0.83	0.75	0.96

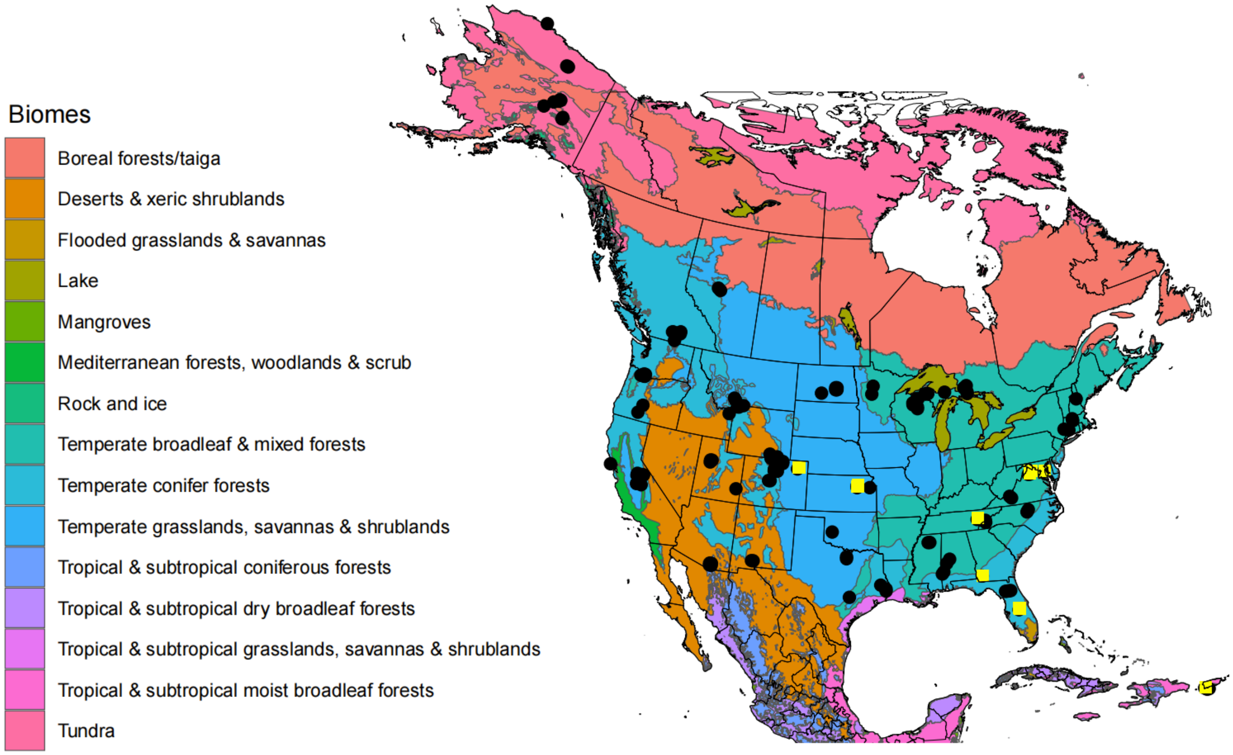

Figure S1 The distribution of the studied 128 operational sites (black dots) among 15 biomes across North America. The yellow squares highlight the nine NEON sites where the 45 human-dominated plots are located.

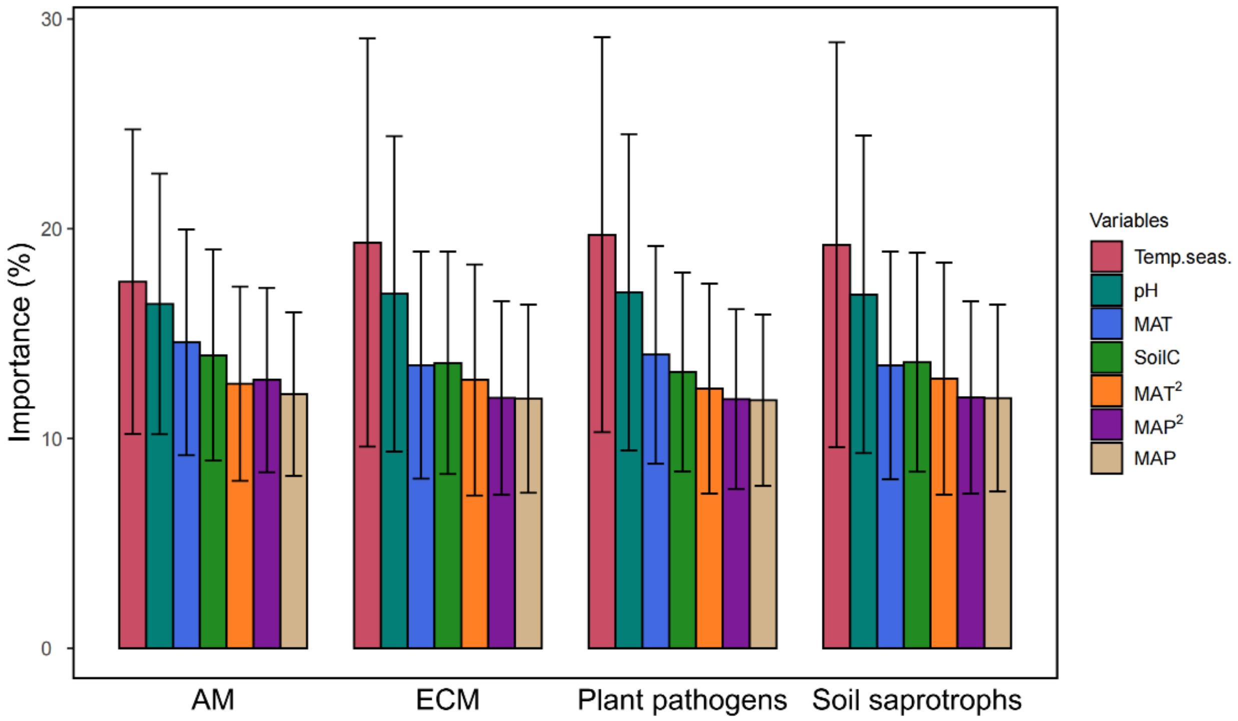

Figure S2 The importance of individual climate and soil variables in predicting guild-level species occurrences. The environmental variables included mean annual temperature (MAT) and its quadratic term (MAT^2), mean annual precipitation (MAP) and its quadratic term (MAP^2), temperature seasonality (Temp.seas.), soil pH (pH), and total soil carbon content (SoilC). Error bars represent the standard deviation of variable importance across species. EM, Ectomycorrhizal mycorrhizal fungi; AM, arbuscular mycorrhizal fungi.

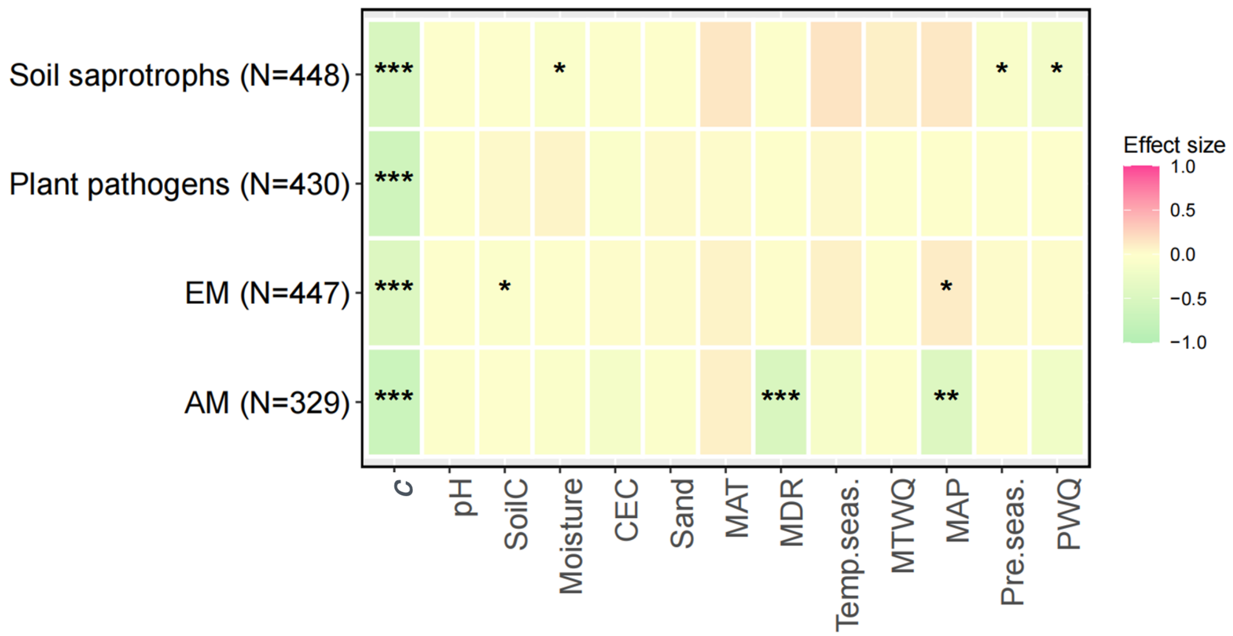

Figure S3 The underlying drivers of the guild-level *z* parameters (i.e., scaling slopes). The linear mixed-effects model included seven climate variables of precipitation of the warmest quarter (PWQ), precipitation seasonality (Pre.seas.), temperature seasonality (Temp.seas.), mean annual precipitation (MAP), mean annual temperature (MAT), mean temperature of the wettest quarter (MTWQ) and mean diurnal range (mean of monthly (max temperature-min temperature); MDR), five soil variable of soil sand content (Sand), cation exchange capacity (CEC), soil carbon content (SoilC), soil moisture and pH, and local fungal diversity (i.e., *c* parameter). The numbers in parentheses represent the number of plots included in the analysis. *P*-values were adjusted to account for multiple comparisons using the Benjamini-Hochberg (BH) approach. EM, Ectomycorrhizal mycorrhizal fungi; AM, arbuscular mycorrhizal fungi. ***$P \leq 0.001$; **$P \leq 0.01$; * $P \leq 0.05$.

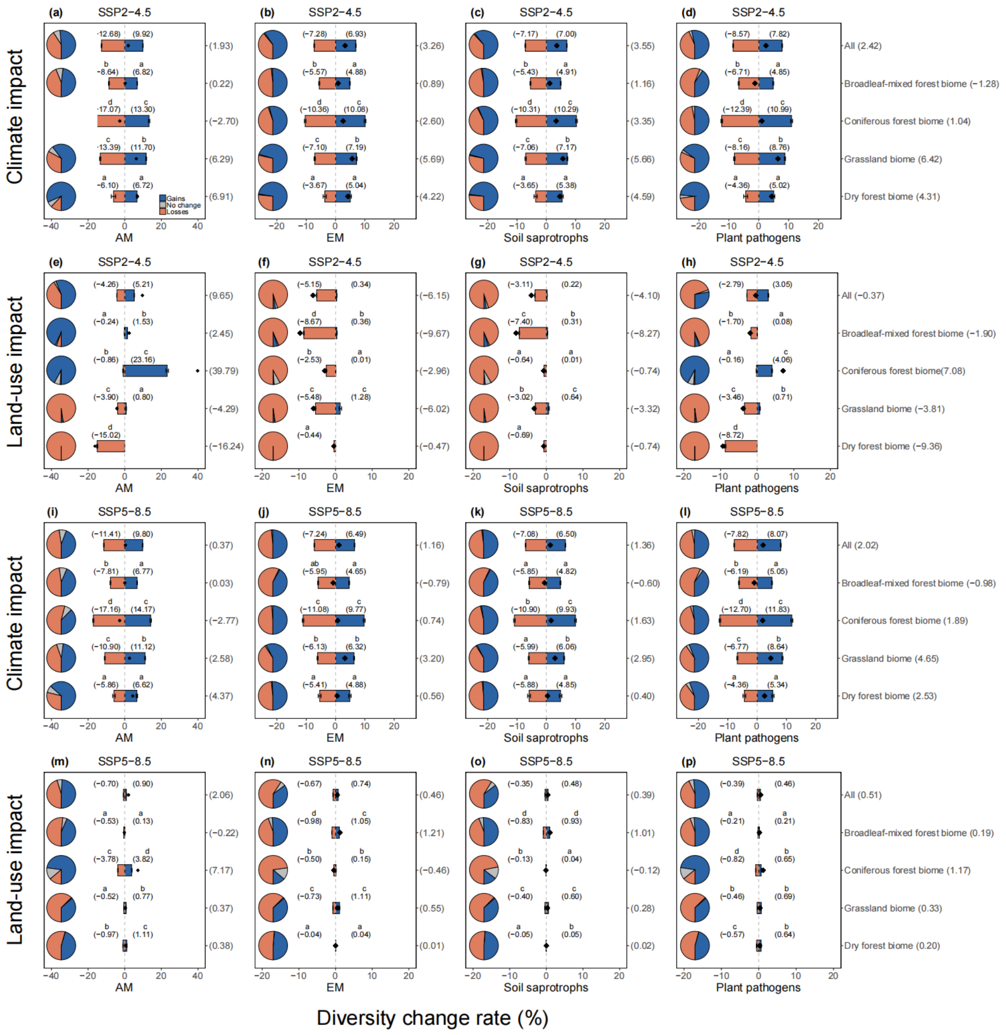

Figure S4 Comparing the guild-level mean diversity loss and gain rates among biomes with diversity losses and gains driven either by climate (a–d; i–l) or land-use change (e–h; m–p) under both moderate- (SSP2–4.5) and high-emission scenarios (SSP5–8.5). For each biome and fungal guild, the bars show both estimated mean diversity loss (coral) and gain (blue) rates (mean ± 95% CI), adjusted for differences in sample sizes and random effects. Bars that share a letter do not differ significantly in their estimated means. The black diamond indicates the net diversity change rate derived from the raw data. For each guild, the mean diversity loss and gain rates were also estimated across biomes (All). The four biomes included temperate broadleaf and mixed forests (Broadleaf-mixed forest biome), temperate coniferous forests (Coniferous forest biome), temperate grasslands (Grassland biome), and savannas and shrublands, and tropical and subtropical dry broadleaf forests (Dry forest biome). EM, Ectomycorrhizal mycorrhizal fungi; AM, arbuscular mycorrhizal fungi.

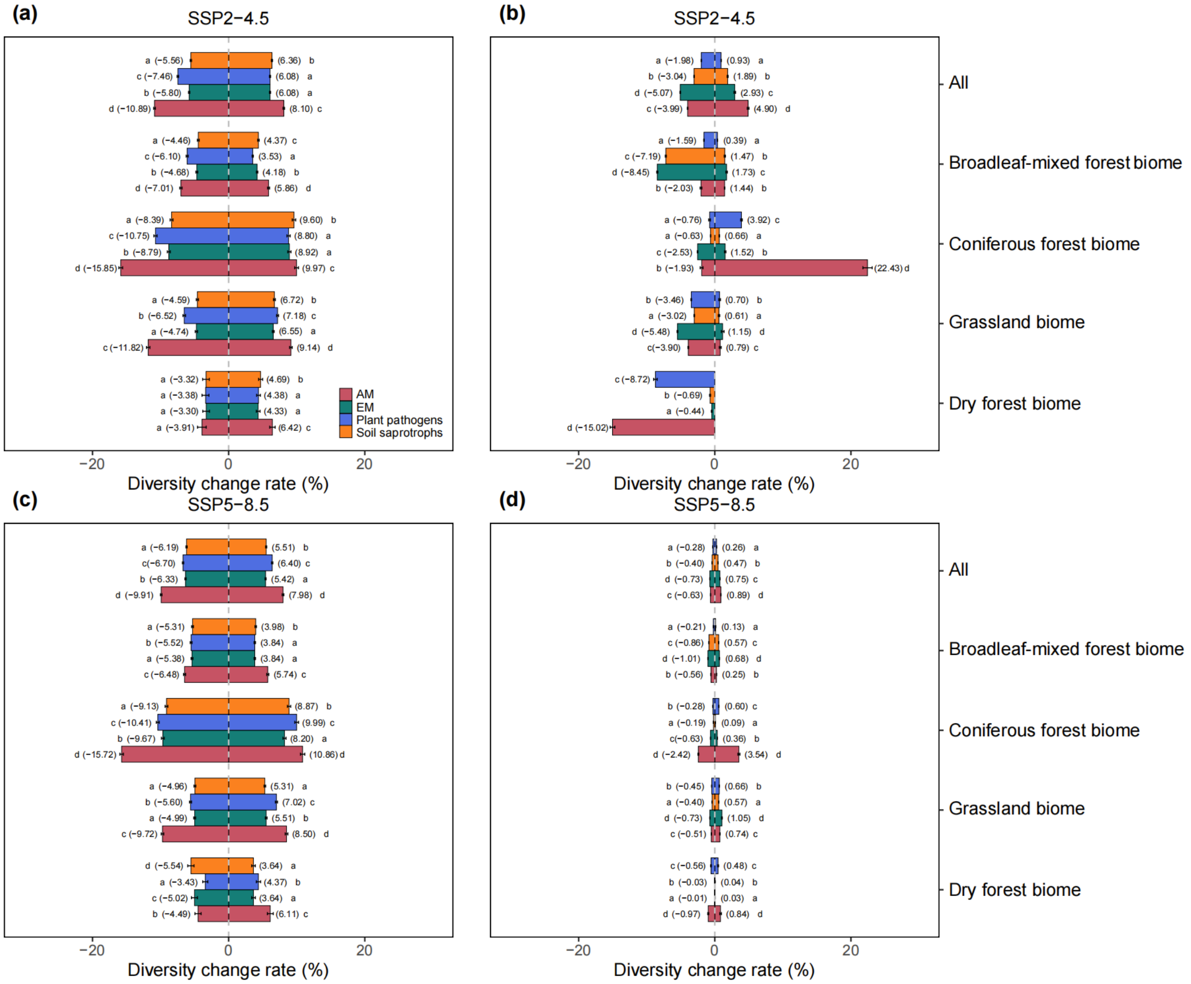

Figure S5 Comparison of the mean diversity loss and gain rates among fungal guilds with diversity losses and gains driven either by climate (a–c) or land-use change (b–d) under both moderate- (SSP2–4.5) and high-emission scenarios (SSP5–8.5). The comparison was performed both within and across biomes (All). The values on the bar represent the estimated marginal means (mean ± 95% CI), adjusted for differences in sample sizes and random effects. Bars that share a letter do not differ significantly in their means. The four biomes included temperate broadleaf and mixed forests (Broadleaf-mixed forest biome), temperate coniferous forests (Coniferous forest biome), temperate grasslands (Grassland biome), and savannas and shrublands, and tropical and subtropical dry broadleaf forests (Dry forest biome). EM, Ectomycorrhizal mycorrhizal fungi; AM, arbuscular mycorrhizal fungi.

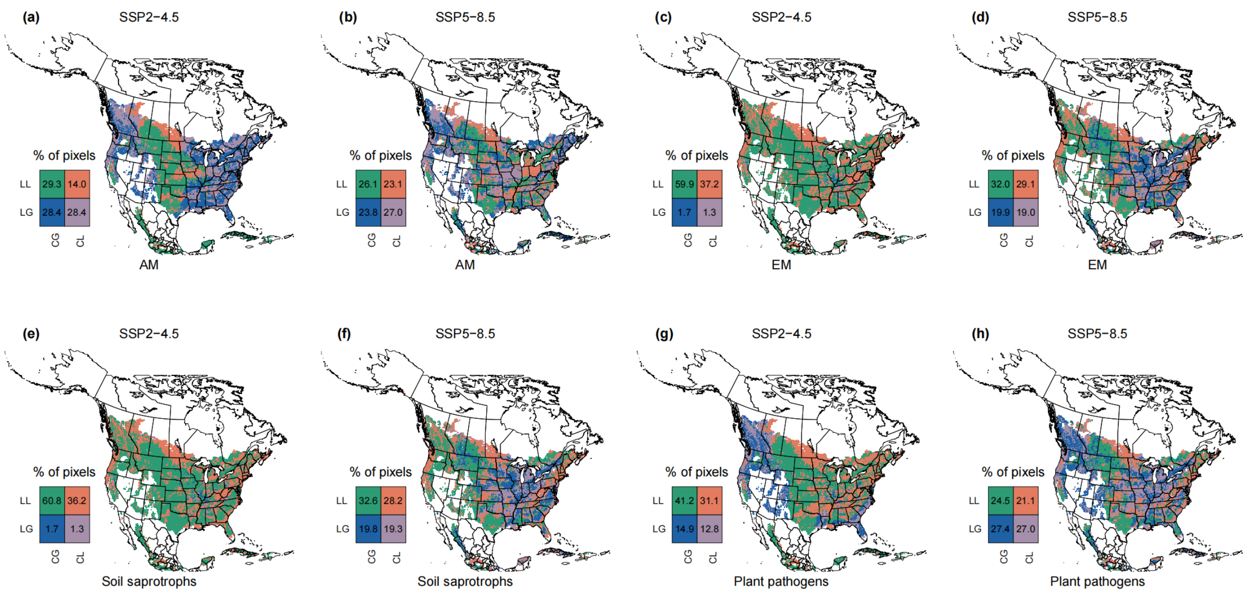

Figure S6 The bivariate map showing the spatial overlap of climate and land-use change effects on the guild-level fungal diversity losses and gains under moderate- (SSP2−4.5) and high-emission scenarios (SSP5−8.5). Pixels in each panel are colored to represent various combinations of climate and land-use change effects, which are classified as either causing diversity losses due to climate change (CL) or land-use change (LL), or diversity gains due to climate change (CG) or land-use change (LG). Numbers in the legend indicate the proportion of pixels that exhibit various combinations of climate and land-use change effects. EM, Ectomycorrhizal mycorrhizal fungi; AM, arbuscular mycorrhizal fungi.

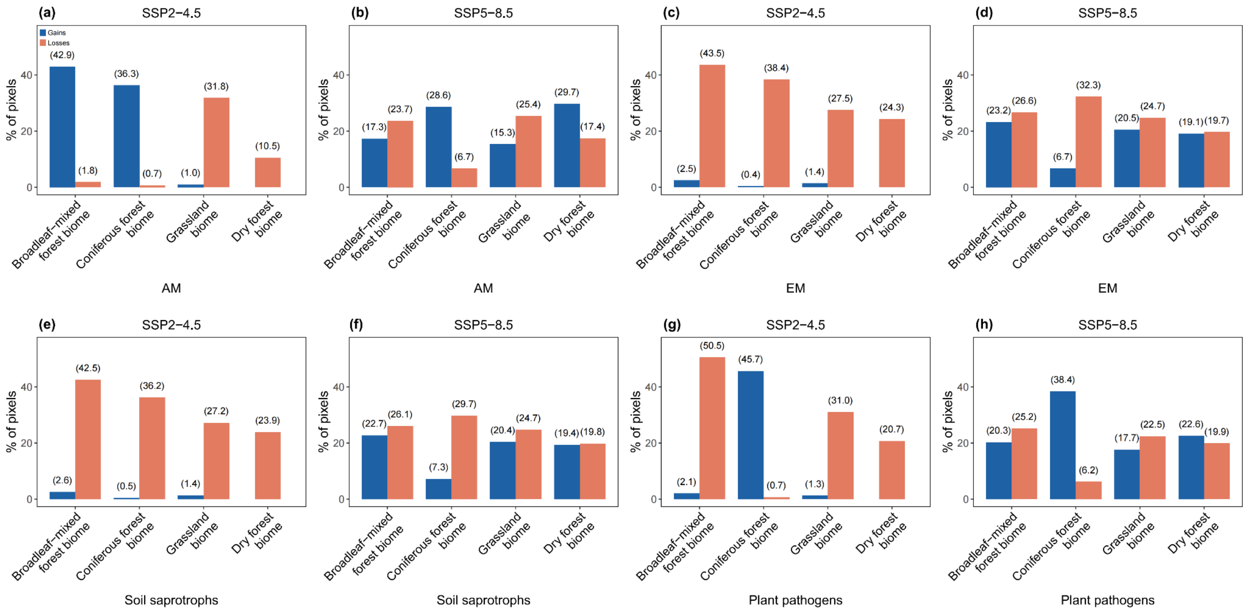

Figure S7 The spatial overlap of climate and land-use change effects on the guild-level fungal diversity losses and gains in each biome under moderate- (SSP2–4.5) and high-emission scenarios (SSP5–8.5). Numbers on the bars represent the proportion of pixels in each biome where both factors contribute to species losses (blue) or gains (coral). The four biomes included temperate broadleaf and mixed forests (Broadleaf-mixed forest biome), temperate coniferous forests (Coniferous forest biome), temperate grasslands (Grassland biome), and savannas and shrublands, and tropical and subtropical dry broadleaf forests (Dry forest biome). EM, Ectomycorrhizal mycorrhizal fungi; AM, arbuscular mycorrhizal fungi.

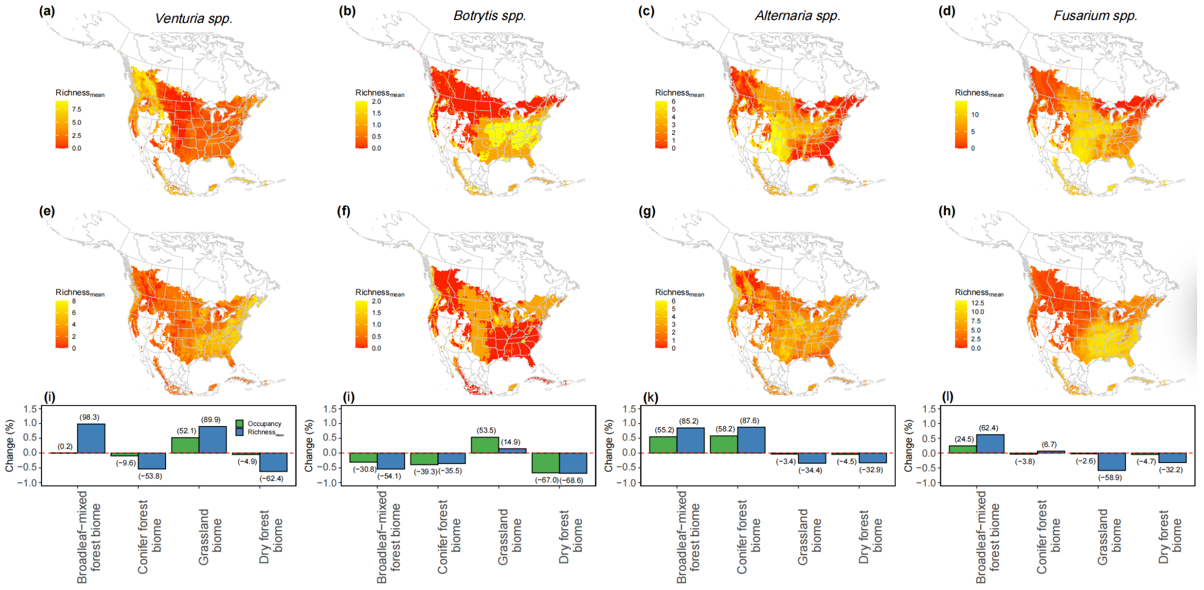

Figure S8 Current and projected diversity (i.e., mean richness per grid cell) and occupancy for four common plant pathogenic fungal genera are presented. Projections are based on the RCP 5-8.5 climate scenario. The third row of panels displays the projected changes in diversity and total occupancy of each genus across four biomes. Changes in diversity and occupancy were calculated as the difference between current and future scenarios, divided by the current metrics. Grid-level mean richness was calculated from the occurrence of individual species within each genus, while occupancy was defined as the total number of grid cells occupied by at least one species in the genus.

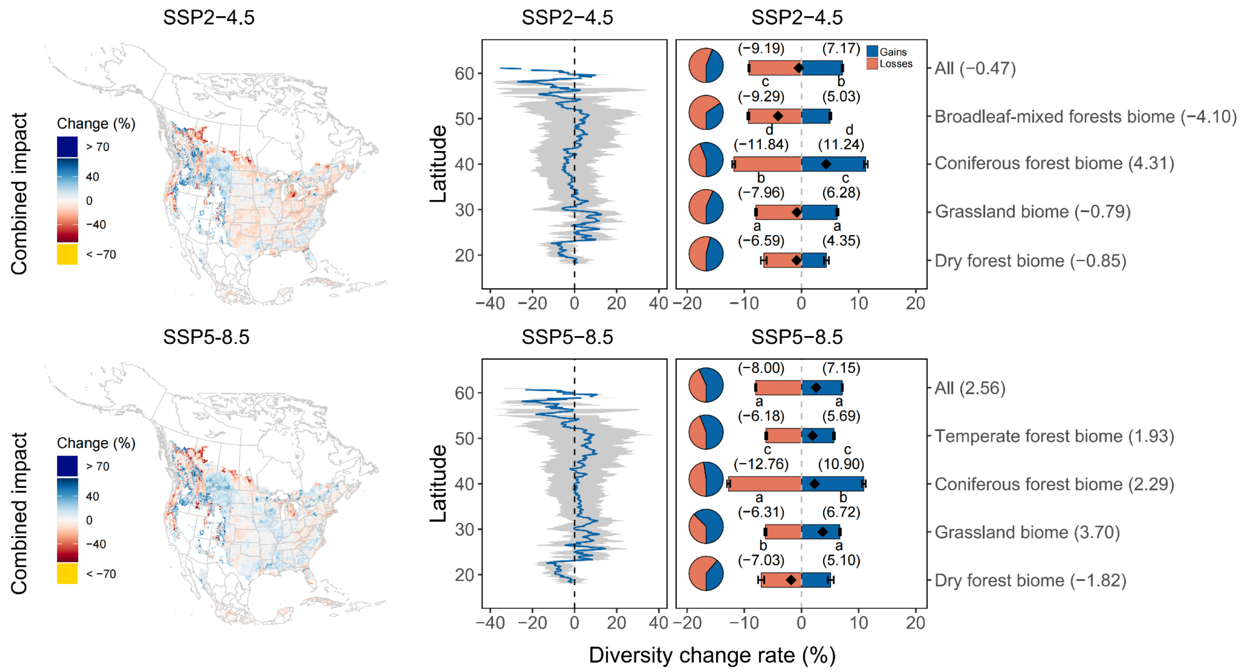

Figure S9 The potentially combined effect of climate and land-use changes on soil fungal diversity change rate for the whole fungal communities under moderate- (SSP2–4.5) and high-emission scenarios (SSP5–8.5). The combined effect of both factors was estimated by summing their individual effect, assuming an additive relationship between them. Panels in the first column show the spatial variation of diversity loss and gain rates; panels in the second column show the net diversity change rate along latitudes (mean ± se). For panels in the third column, the pie charts show the proportion of pixels predicted to undergo diversity losses, gains, or no change. The coral and blue bars indicate the estimated mean diversity loss and gain rates (mean ± 95% CI), respectively. The black diamonds, with their values displayed on the right y-axis, indicate the net diversity change rate determined based on the raw data. Diversity loss and gain rates were estimated for each biome of temperate broadleaf and mixed forests (Broadleaf-mixed forest biome), temperate coniferous forests (Coniferous forest biome), temperate grasslands (Grassland biome), savannas and shrublands, and tropical and subtropical dry broadleaf forests (Dry forest biome) and across four biomes (All). The letters on the bars represent differences in the mean diversity loss and gain rates among biomes. Bars that share a letter do not differ significantly in their means. Estimated means across biomes are presented to summarize the general pattern and were not compared to any biome-specific estimates.

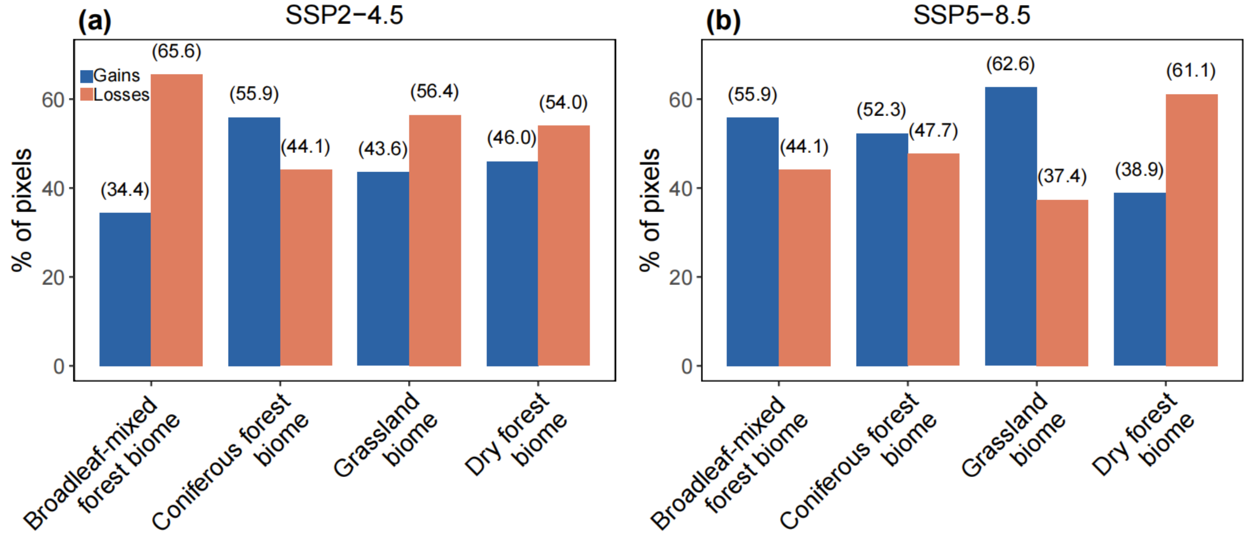

Figure S10 The predicted spatial extent of diversity losses and gains driven by the potentially combined effect of climate and land-use changes under moderate- (SSP2–4.5) and high-emission scenarios (SSP5–8.5). The combined effect of both factors was estimated by summing their individual effect, assuming them being additive. Numbers on the bars represent the proportion of pixels in each biome predicted to experience diversity losses (coral) or gains (blue). The four biomes included temperate broadleaf and mixed forests (Broadleaf-mixed forest biome), temperate coniferous forests (Coniferous forest biome), temperate grasslands (Grassland biome), and savannas and shrublands, and tropical and subtropical dry broadleaf forests (Dry forest biome).

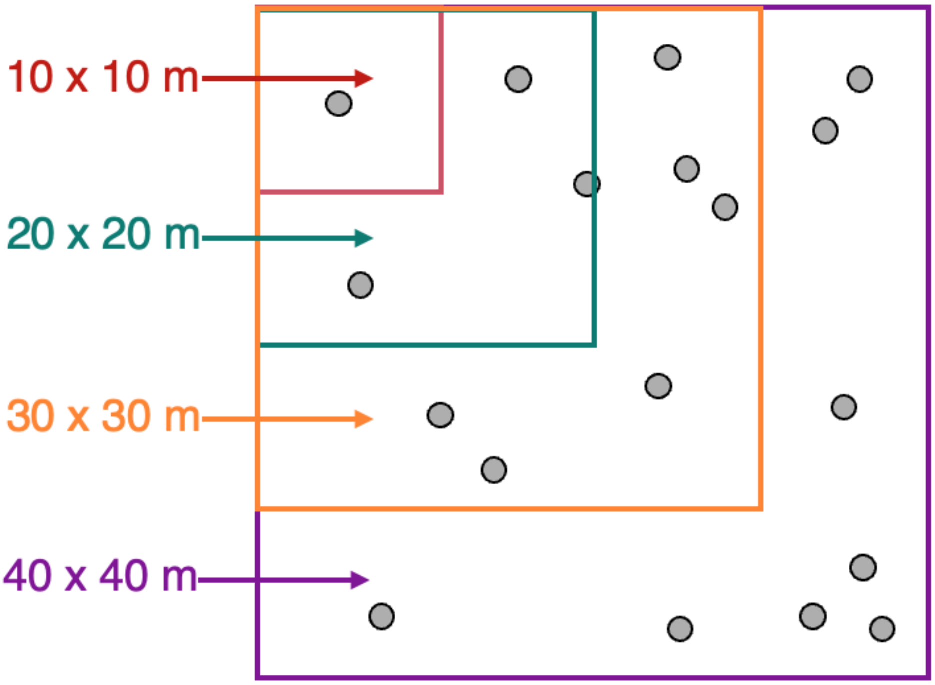

Figure S11 A schematic diagram showing the construction of a plot-scale species–area relationship. Based on the available soil samples and the need to standardize sampling efforts, we estimated total fungal taxonomic diversity at four sampling scales of 100, 400, 900, and 1,600 m^2 by randomly sampling one, four, nine, and 16 soil samples at each scale, respectively.

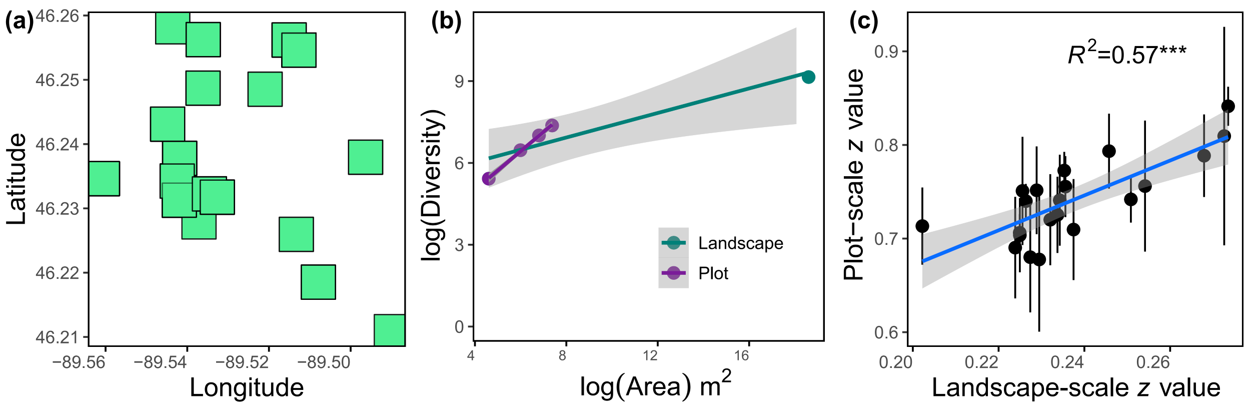

Figure S12 Determination of the site-scale species–area relationship (SAR) and the relationship between scaling slopes quantified at the plot (local) and landscape scales, both for an illustrative single site and across multiple sites. Panel (a) illustrates the distribution of 17 40 × 40 m plots within the University of Notre Dame Environmental Research Center NEON site (UNDE). Panel (b) depicts the plot- and landscape-scale SARs. Taking a plot as an example, the four purple dots in panel (b) represent the mean values of the subplot and plot-scale total fungal taxonomic diversity, while the green dot corresponds to the site-scale diversity estimated based on all soil samples collected from the site. Panel (c) shows the relationship between the plot- and landscape-scale scaling slopes. Error bars are shown only for the plot-scale data, representing the standard deviation of scaling slopes across plots, whereas only one scaling slope (i.e., *z* value) could be determined for each site.

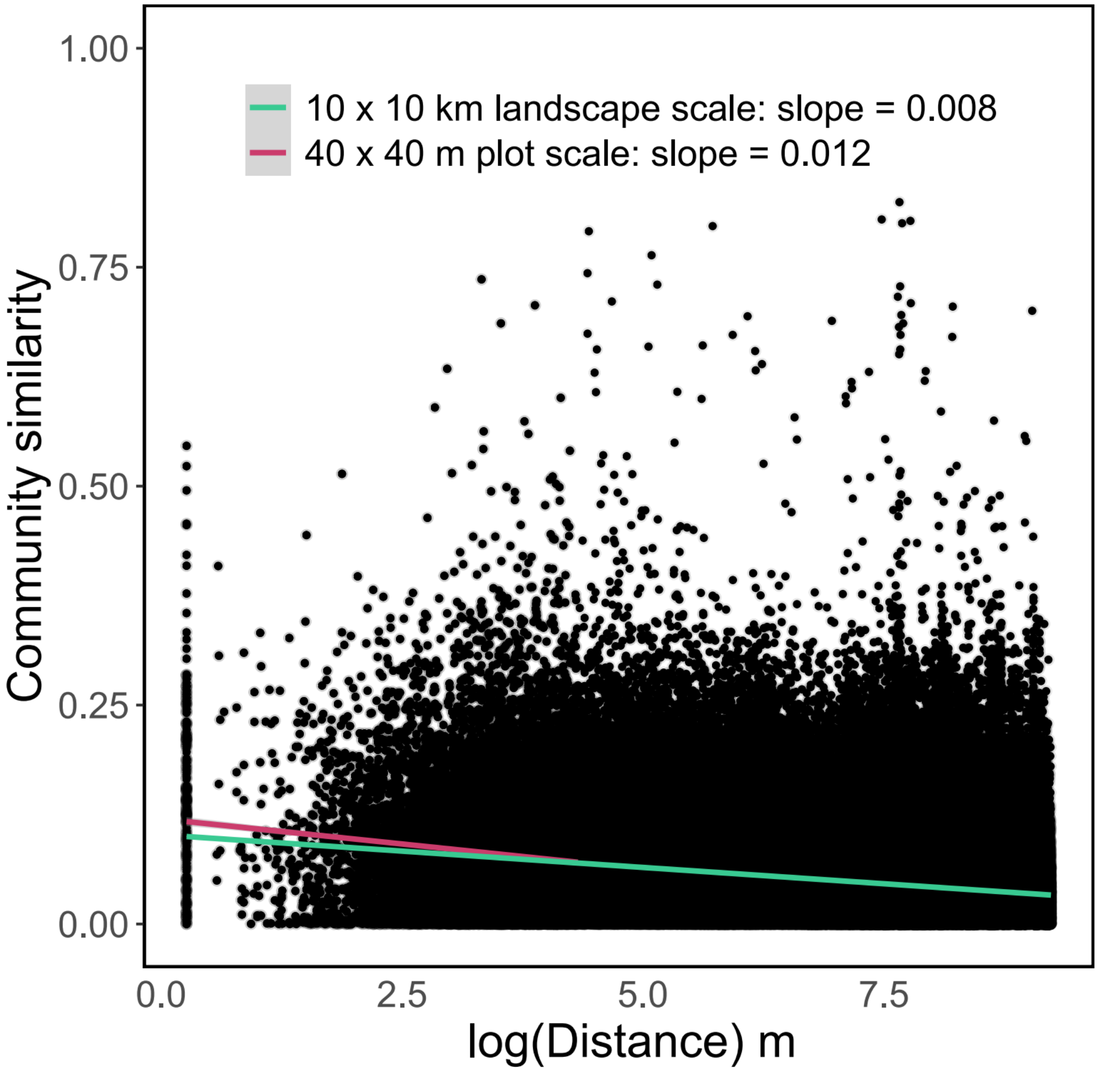

Figure S13 Comparison of the distance-decay pattern of soil fungal communities examined at the 40 × 40 m plot and 10 × 10 km landscape scales.

Supplementary Note 1 Determination of the historical land-use type for NEON plots.

The land-use data represents the history of land-use and land-cover data for the conterminous United States at an annual timescale and 1 × 1 km spatial resolution in the past 390 years (1630–2020). Each pixel is classified into one of six land-use types: urban, cropland, pasture, forest, shrub, or grasslands. The historical land-use type for each NEON plot was determined based on plot coordinates. Since the historical land-use type for a plot could change over the 390-year period, we defined its baseline historical land-use type as the most frequently occurring type over time.

We compared fungal diversity between each of the 45 human-dominated plots and their adjacent natural plots, ensuring that the paired plots shared the same historical land-use type, corresponding to the current land-use type of the natural plots. For instance, if a human-dominated plot had been converted from forest, fungal diversity in the plot was compared to that of adjacent natural plots that had remained forested both historically and currently (Table S1). This comparison approach also indicates that diversity in natural plots was assessed not from specific individual plots but from a collection of multiple plots with similar historical and current land-use types.

Paired plots were generally located within the same NEON site or, when no within-site analogous natural plots were available, within the same biome. Multiple natural plots were typically selected as the historical land-use data proved a broad (e.g., forest) rather than an explicit definition of land-use type (e.g., evergreen forest or mixed forest). In that case, if the historical land-use type of a human-dominated plot was forest, its paired natural plots could be evergreen, deciduous, or mixed forest. This practice aligns with the study goal of understanding how land-use change from natural to human-dominated land systems affects fungal communities. As described in the method sections 2.3 and 2.4, a rarefaction approach was used to estimate fungal diversity to account for differences in sample size among paired plots.

Supplementary Note 2 Derivation of species' relative habitat affinity for the countryside SAR. The countryside SAR framework accounts for the conservation value of countryside landscapes by introducing a parameter h_j, reflecting the relative affinity of a species group to human-dominated habitat type j compared to its original habitat. Assuming that affinity to the original habitat is one (which is a matter of standardization), h_j reflects the proportion of area that can be effectively used by the species group. For example, a habitat affinity of 0.2 means that a unit area of modified habitat would be of the same quality as 20% of a unit area of the original habitat. The countryside SAR framework is:

$$S = c\left(\sum_j h_j A_j\right)^z$$

with h_j indicates species relative affinity to land-use type j, i.e., proportion of area that can be effectively used by the species group. Changes in species taxonomic diversity after transforming area A with known composition to area A_0 with different compositions can be estimated as:

$$\frac{S_j}{S_{j\,orig}} = \left(\frac{\sum_j h_j A'_j}{\sum_j h_j A_j}\right)^z$$

Assume complete transformation from one unit area of original habitat to one unit area of modified habitat j, i.e., A_{orig}= 1 and A_j= 0 to A'_{orig} = 0 and A'_j = 1. Then the change in species diversity could be estimated as:

$$\frac{S_j}{S_{j\,orig}} = \left(\frac{h_{orig}A'_{orig}+h_jA'_j}{h_{orig}A_{orig}+h_jA_j}\right)^z$$

$$= \left(\frac{h_jA'_j}{h_{orig}A_{orig}}\right)^z$$

$$= \left(\frac{h_j}{h_{orig}}\right)^z$$

Assume that the species group has affinity = 1 for the original habitat (which is just a matter of standardization), then habitat affinity can be expressed as the ratio of species diversity before and after habitat modification, powered by the SAR scaling slope as follows:

$$h_j = \left(\frac{S_j}{S_{j\,orig}}\right)^{\frac{1}{z}}$$

The ratio $\frac{S_j}{S_{j\,orig}}$ is a so-called response ratio (RR) of species richness S_j in the modified and $S_{j\ \mathrm{orig}}$ in the original (semi-) natural habitat. Hence, we can express the habitat affinity as a response ratio powered by the SAR scaling slope.

$$h_j = \left(\frac{S_j}{S_{j\,orig}}\right)^{\frac{1}{z}} = RR^{\frac{1}{z}}$$

Supplementary Note 3 Testing the generalization of plot-scale scaling slopes to the landscape scale.

We used two approaches to test if and to what degree the plot-scale scaling slopes could be generalized to the landscape scale. First, we fitted a landscape-scale SAR for 21 NEON sites within which all plots could roughly be encompassed by a 10 × 10 km rectangle, a comparable scale where the species distribution model was constructed. We then determined the subplot and plot-scale fungal total taxonomic diversity as described in the method section 2.4 and the site-scale diversity with extrapolation based on the available soil samples. Finally, we estimated the site-scale SAR and scaling slopes based on the fungal diversity estimated at the 100, 400, 900, 1,600 m^2 and 100 km^2 spatial scales (Fig. S12a, b).

The results showed that the site-scale scaling slopes were highly correlated with the plot-scale scaling slopes (Fig. S12c), suggesting that the diversity scaling relationship between these two scales is tightly coupled. Although the landscape-scale scaling slopes appeared to be lower than the plot-scale values, they may have been underestimated due to an underestimation of the site-scale diversity. Specifically, based on the plot-scale sampling intensity, approximately 10×10^7 soil samples would be required to estimate the site-scale diversity. Consequently, it is highly possible that the site-scale scaling slopes are closer to the plot-scale values than we observed here. For the second approach, we quantified the distance-decay patterns of soil fungal community similarity at the 40 × 40 m plot scale and the 10 × 10 km landscape scales, assuming that these patterns would reflect how diversity scales with area as previously proposed. We found that the distance-decay slopes estimated at both scales were largely similar, approximately 0.01 (Fig. S13). Based on these observations and considering that our study focused on diversity change rates over time (i.e., a relative value), where the potential impact of scaling slopes on diversity estimation would be minimized, we deemed it feasible to use the plot-scale scaling slope to parameterize the countryside SAR framework.

Supplementary Note 4 Testing the difference in mean diversity loss and gain rates among biomes and fungal guilds.

To assess the susceptibility of different fungal guilds to climate and land-use changes, we compared the mean diversity loss and gain rates among guilds within each biome and across biomes with a generalized linear mixed-effects model. For within-biome comparisons, guild-level diversity loss or gain rates were treated as the response variables, while fungal guild, biome type, and their interaction term were included as fixed effects, with pixel identity as a random effect. For cross-biome comparisons, the fungal guild was included as a fixed effect, with pixel identity as a random effect. Pairwise differences in mean values were computed using the *emmeans* function from the "*emmeans*" package .

To better understand the vulnerability of different biomes to climate and land-use changes, we compared the mean diversity loss and gain rates among biomes for both the whole community and individual guilds. In both cases, we used a generalized least squares (GLS) model to account for autocorrelation. The model included raw diversity loss and gain rates as response variables, with biome type as the explanatory variable. To reduce data skewness, we applied a logarithmic transformation to diversity gain rates and a square root transformation to the absolute values of diversity loss rates, and reported the estimated means after back-transforming the data.
